# Supplementary material for: Workplace interventions to prevent suicide: A scoping review
Source: PLoS One. 2024 May 2;19(5):e0301453. doi: 10.1371/journal.pone.0301453 (PMC11065308; doi:10.1371/journal.pone.0301453)
Supplement: S1 File — (DOCX) [file pone.0301453.s002.docx]

**MEDLINE search strategy**

Ovid MEDLINE(R) ALL <1946 to March 20, 2023>

1 Suicide/ or suicide.mp. 97681

2 "suicide prevention".mp. 14835

3 Workplace/ or workplace.mp. 62995

4 organi?ation.mp. 883519

5 Workforce/ or workforce.mp. 107524

6 intervention.mp. 797982

7 program?.mp. 914610

8 response.mp. 2841616

9 strateg?.mp. 693493

10 1 or 2 97681

11 3 or 4 or 5 1014152

12 6 or 7 or 8 or 9 4872085

13 10 and 11 and 12 1205

14 6 or 7 or 9 2223829

15 10 and 11 and 14 1049

16 limit 15 to (english language and yr="2002 -Current") 788
