# Supplementary material for: Workplace interventions to prevent suicide: A scoping review
Source: PLoS One. 2024 May 2;19(5):e0301453. doi: 10.1371/journal.pone.0301453 (PMC11065308; doi:10.1371/journal.pone.0301453)
Supplement: S1 Table — (DOCX) [file pone.0301453.s003.docx]

**Supplementary table 1. Study characteristics**

| **Citation [ref in main text]**  **Country** | **Sector**  **Profession** | **Design** | **Intervention** | **Intervention components** |
| --- | --- | --- | --- | --- |
| Rozanov et al (2002) [53]  Ukraine | Military  Army | Cohort study | Prevention programme | 1. Seminar for professional officers (medical doctors, psychologists, educational officers). 5 days on: 1) basic knowledge on suicide and basic skills in prevention; 2) specific aspects of suicide and special skills of suicide prevention; 3) development of the specific suicide prevention strategy in the specific military environment. 2. Targeted training for basic soldiers, esp. conscripts. Weekly basis for 4 months. Basic skills in identifying suicide problems in the military community. 3. 3-day gatekeeper training for professionals and unit commanders. 4. Distribution of booklets for all soldiers on suicide prevention. |
| Knox et al (2003) [51]  USA | Military  Air force | Cohort study | United States Air Force Suicide Prevention Programme (USAFSPP) | 1. Leadership involvement 2. Suicide prevention education within training 3. Guidelines for commanders (improve referrals for MH evaluation by emphasising that commanders and MH professionals are partners in improving duty performance) 4. Community preventive services 5. Community education and training 6. Investigative interview policy (individuals under investigation to be assessed for suicide risk) 7. Critical incident stress management (CISM team to respond to traumatic events) 8. Integrated delivery system for human services prevention (to provide centralised information and referral and decision-making) 9. Limited patient privilege (therapist-patient privilege for individuals at risk of suicide to promote help-seeking behaviour) 10. Behavioural health survey 11. Suicide event surveillance system |
| Joseph et al (2004) [69]  India | Military  Air force | Intervention case study | Institute of Aerospace Medicine (IAM) awareness training | 1. Mentor system for trainees 2. Buddy system (to monitor progress and behaviour of trainees) 3. IAM awareness training |
| Halliwell and Hoskin (2005) [56]  UK | Veterinary surgeons | Descriptive | Various | 1. Helpline 2. Psychiatric assessment and care |
| Garelick et al (2007) [34]  UK | Healthcare  Doctors | Cross-sectional | MedNET Service for Doctors | 1. Self-referral service staffed by 3 consultant psychiatrists in psychotherapy. |
| Gordana and Milivoje (2007) [68]  Serbia and Montenegro | Military  Army | Cohort study | Suicide prevention program | 1. Soldier/professional friend to recognise signs, included within training - to identify others  2. Primary MH team within the unit  3. Secondary MH team in the medical centre.  4. Tertiary MH team to refer. |
| Nakao et al (2007) [25]  Japan | Office workers | Cohort study | Employee Assistance Programme (EAP) | 1. Counselling via phone or email (free and anonymous) 2. Referral to psychiatric clinic affiliated with the institute (health insurance coverage) 3. Seminar for works x5 on 1) self-care; 2) knowledge of job stress and approaches for coping with stress; 3) effect of excessive work on health; 4) early detection of distressed colleagues; 5) occupational maladjustment |
| Rø et al (2007) [35]  Norway | Healthcare  Doctors | Cohort study | Villa Sana | 1. Single day counselling lasting 6-7 hours  2. Weeklong course with boarding, for 8 individual doctors or 4 couples with lectures and individual counselling. |
| Lapenaite and Vaicaitiene (2008) [54]  Lithuania | Military  Army | Intervention case study | Program of Psychological Support | 1. Psychological training and education about suicide behaviour identification, stress, depression, suicide prevention and intervention and support during a crisis, for military leaders, officers and conscripts.  2. Assessment and identification (monitoring psychological wellbeing by psychological support professionals or medics) |
| Steyn (2008) [63]  South Africa | Emergency services  Police | Cohort study | Suicide prevention workshops | 1. Suicide prevention workshops aimed at destigmatising suicide, increasing awareness of indicators of suicidal behaviour and teaching how to manage a suicidal person (and how to manage stress and stress reactions) 2. Generic stress management training by social workers 3. Critical incident debriefing 4. Psychometric recruitment criteria |
| Brown (2010) [60]  UK | Veterinary surgeons | Description | Various | 1. Vet Helpline 2. Veterinary Surgeons' Health Support Programme 3. Vetlife website |
| Knox et al (2010) [57]  USA | Military  Air Force | Cohort study | USAFSPP | As above (Knox et al, 2003) but:  7. Trauma stress response (Trauma stress response teams established to help personnel deal with the emotions they experience in reaction to traumatic incidents) and 9. Afforded increased confidentiality when seen by MH providers |
| Levenson et al (2010) [58]  USA | Emergency services  Police | Intervention case study | Badge of Life Psychological Survival for Police Officers Program (BOL) | 1. Emotional Self-Care training programme designed to focus on the ability to care for their own emotional wellbeing (resilience)  2. Annual mental health checks  3. Peer support officers to conduct the training, set the example and encourage involvement |
| Dwyer et al (2011) [52]  Australia | Healthcare  Junior Doctors | Intervention case study | Prevention programme | 1. Safe-hours rostering to protect work-life balance 2. Effective supervision 3. Open, two-way communication with management 4. 24-hour on-call peer support program with peers training in counselling and HR policies on bullying, harassment and equal opportunities. 5. Identification of poorly performing Drs 6. Procedure for managing poorly performing or at-risk medical staff 7. External referral as needed. |
| Gullestrup et al (2011) [38]  Australia | Construction industry | Cohort study | MATES in Construction | 1. General awareness training 2. Connector (a mate who can keep you safe while connecting you to help) training 3. Suicide first aid 4. Field officers 5. Case management 6. Suicide prevention hotline 7. Specialist intervention (field officers/case managers assess and refer at-risk workers to external services) 8. Postvention |
| Warner et al (2011) [36]  USA | Military  All | Cohort study | Suicide prevention program | 1. Recognition and response training for platoon level soldiers (30-50) in risk factors, EWS, promoting assistance-seeking behaviours.  2. Spouse's Battlemind Training Prog. Encouraged participation of family members.  3. Specific sessions for leaders on managing emergent situations.  4. Recognition and early management of stress, PTSD, sleep etc. by chaplain's, MH personnel etc.  5. Suicide prevention review board and suicide risk management teams  6. Unit behavioural health needs assessment to monitor unit-wide MH issues (morale, cohesion etc.)  7. Applied Suicide Intervention Skills Training (ASIST)  8. Incident response and trend monitoring  9. Post-deployment health assessment and risk stratification.  10. Those screened at high risk received education and support. |
| Mishara and Martin (2012) [48]  Canada | Emergency services  Police | Cohort study | Together for Life (*Ensemble pour la vie*) | 1. Training for all police units - half day, on nature of suicide, identification of suicide risk and how to help a colleague in difficulty.  2. Telephone helpline - leave message and called back by a police volunteer trained in suicide prevention 'in complete discretion'.  3. Supervisor and union representative training - to identify at risk officers and how to provide help  4. Publicity campaign to inform officers about suicide prevention |
| Downs et al (2014) [33]  USA | Healthcare  Medical Students | Cross-sectional | Healer Education Assessment and Referral (HEAR) | 1. Reduce stress in the workplace (small and large modifications suggested) 2. Increase awareness of risk factors for suicide (recognising warning signs, encouraging help-seeking behaviour and offering hope, referring for help) 3. Foster utilisation of MH support services and resources |
| Doran et al (2015) [40]  Australia | Construction industry | Cohort study | MATES in Construction | 1. GAT for workers 1-hr session  2. Connector training for workers who volunteer. Trained to identify and safely engage with people at risk and connect them to professional help  3. ASIST for key workers to make a safe plan for a person at risk and connect them to external resources  4. Support provided through field officers, case managers and a suicide prevention hotline |
| Finney et al (2015) [64]  USA | Emergency services  Fire Service | Intervention case study | Houston Fire Department (HFD) model | 1. Raising awareness 2. Education about suicide - for firefighters and managers |
| Shelef et al (2015) [46]  Istrael | Military  Army | Intervention case study | Israeli Defence Force (IDF) Suicide Prevention Program (SPP) | 1. Personal weapons locked in storage when on leave  2. Revision of old and development of new procedures and commands to improve screening and management of suicidal soldiers  3. 'There is a way' psycho-educational curriculum for soldiers (especially commanders of all ranks) to detect and identify symptoms of MI or overwhelming stress, and how to deal with it.  4. Mutual responsibility programme designed to teach commanders to reduce stigma related to help-seeking from professionals when in distress, and to educate soldiers about mutual responsibility for their own lives and their peers'.  5. MH officers integrated into various army units to reduce stigma. |
| Doran et al (2016) [28]  Australia | Construction industry | Cohort study | MATES in Construction | 1. General awareness training (GAT)  2. Connector training  3. Applied suicide intervention skills training (ASIST) |
| Ey et al (2016) [59]  USA | Healthcare  Medical residents and fellows | Feasibility study | Resident and Faculty Wellness Program | 1. Wellness promotion workshops  2. Orientation presentations about the program and referral processes to trainees, chief residents and program directors  3. Suicide prevention screening offered  4. Monthly support groups/luncheons  5. Individual counselling  6. Psychiatric evaluation/meds management  7. Referral to specialised services as needed |
| Martin et al (2016) [37]  Australia | Construction industry | Cohort study (5-year follow-up) | MATES in Construction | 1. General awareness training (GAT)  2. Connector training  3. Applied suicide intervention skills training (ASIST)  4. Field officers  5. Case management  6. 24-hour suicide prevention telephone line  7. Postvention |
| Shelef et al (2016) [29]  Israel | Military  Army | Cohort study | IDF SPP | See Shelef et al, 2015 |
| Wentworth (2016) [30]  USA | University | Quasi-experimental (PhD thesis) | Question, Persuade, Refer (QPR) gatekeeper training | 1. Short training programme (1-1.5 hrs) teaching how to engage with an individual demonstrating signs of suicidality or a MH crisis |
| National Mental Health Commission (2017) [50]  Australia | Military  All | Review | Services involved in the prevention of suicide and self-harm for Australian Defence Force members | 1. Information to raise awareness  2. Training seminars and workshops  3. BattleSMART resilience training program  4. Screening programmes  5. Clinical treatment services  6. ADF rehabilitation programme  7. Drug and alcohol management and treatment  8. Transition support services  9. Post-discharge GP health assessments  10. Health and wellbeing programmes for former-serving members  11. Defence Families Australia providing advocacy services for family members |
| Arnold (2018) [65]  USA | Healthcare  Resident physicians | Intervention case study | Wellness curriculum | 1. 17-module curriculum for self-care, recognising suicide risks in self, dealing with medical errors etc. |
| Davidson et al (2018) [31]  USA | Healthcare  Nurses | Cross sectional | Healer Education Assessment and Referral (HEAR) | 1. Didactic presentations on burnout, depression and suicide, destigmatising depression (awareness)  2. Web-based screening and assessment to identify and refer those at risk of depression or suicide  3. Counselling  4. Referrals |
| King et al (2018) [42]  Australia | Construction industry | Pre- and post-test study | Universal General Awareness Training (GAT) | 1. 1-hour GAT training provided by MATES staff either standalone session or a component of the Life Skills Toolbox, a training programme for apprentices. |
| Kubo et al (2018) [71]  Japan | Office workers | Pre- and post-test study | Mental health first aid (MHFA) | 1. 2-hr MHFA training for some workers |
| King et al (2019) [26]  Australia | Construction industry | Pre- and post-test study | MATES in Construction: General Awareness Training (GAT) | 1. 1-hour GAT training to engage and activate construction workers in suicide prevention. |
| Ramchand et al (2019) [61]  USA | Emergency services  Police | Cross-sectional | Current practices | 1. Minimal services: no prog. beyond the presence of a municipal EAP and/or health insurance  2. Basic services: Minimal services plus any of: MH services specific to law enforcement outside of the EAP; process for responding to staff exposed to critical incidents; training on stress/wellbeing  3. Proactive services: minimal and basic services and at least on additional proactive approach to either identify people at risk for suicide or MH problems and/or facilitate them into care inc: process for proactively identifying people at risk; in-house MH care; embedded chaplains within workforce; substance abuse services specific to law enforcement personnel; peer support programme; official health/wellness program.  4. Integrated services: minimal, basic and proactive plus suicide prevention/mental health promotion integrated into day-to-day operations inc: units focused on MH or suicide prevention, or adopting policies to improve officer MH (i.e. restorative sleep policies). |
| Ross et al (2019) [27]  Australia | Construction industry | Pre- and post-test study | MATES in Construction | 1. GAT for workers 1-hr session  2. Connector training for workers who volunteer. Trained to identify and safely engage with people at risk and connect them to professional help  3. ASIST for key workers to make a safe-plan for a person at risk and connect them to external resources  4. Support provided through fiel dofficers, case managers and a suicide prevention hotline |
| Accardi et al (2020) [45]  USA | Healthcare  Nurses | Cohort study | Healer Education Assessment and Referral (HEAR) | See Davidson et al, 2018 above |
| Albott et al (2020) [62]  USA | Emergency services  Emergency healthcare workers | Intervention case study | Battle buddies | 1. Individuals paired 'battle buddies' by clinical area of practice, responsibilities etc.; intention to create daily conversations between pairs to foster a sense of connectedness, validation, support, trust and useful feedback  2. Unit-level support - endorsement of leadership, internal champion, small group sessions (anticipate-plan-deter model)  3. Individual support - free 1:1 MH consultant with no medical record entry; referrals made as needed |
| Ross et al (2020a) [39]  Australia | Construction industry | Longitudinal cohort study | General Awareness Training (GAT) compared with MATES Awareness Training (MAT) | 1. GAT: 1-hr session delivered to all construction workers on large to medium worksites by 2 trainers to groups of 20-300 workers. Taught how to identify warning signs for suicidality and encouraged to offer active support to struggling co-workers.  2. MAT: 15-minutes delivered by a single trainer with up to 20 workers, conversational style with no audio/visual tech. |
| Ross et al (2020b) [43]  Australia | Energy sector | Pre- and post-test study | MATES in Energy General Awareness Training (GAT) | 1. 1-hour GAT session |
| Willson et al (2020) [32]  USA | Healthcare  Student pharmacists | Pre- and post-test study | Suicide Prevention for Pharmacy Professionals (SPPP) | 1. 1-hr large group session with a 2-hr lab session. Aimed at patients and peers/colleagues |
| Won et al (2020) [55]  South Korea | Emergency services  Fire service | Cross-sectional | MH promotion programme | MH evaluation process to identify high-risk firefighters:  1. General education session for all (60 min lecutre and Q&A)  2. 30-min individual counselling for all (emotional support and psychoeducation)  3. Brief intensive counselling (60-min) for high-risk participants and volunteers |
| Adamouski-Marion (2020) [77]  USA | Healthcare  Clinical and nonclinical team | Plan, Do, Study, Act (PDSA) evaluation | Peer support programme: Compassion, Action, Resilience, Empathy (CARE) | 1. Peer support workshops |
| Baker et al (2021) [66]  USA | Military | Intervention case study | The Airman’s Edge Project (peer to peer support programme) | 1. Curriculum-based education and information provided by peer mentors as a group intervention (on unit cohesion, purpose and morale; sleep quality; firearm storage)  2. Provision of encouragement and informal types of support by peer mentors as a dyadic or 1:1 intervention (the peer support model)  3. Peer mentors trained in crisis response planning |
| Doran et al (2021) [41]  Australia | Construction industry | Cohort study | MATES in Construction: Case management model | Case manager:  1. Help clients identify needs  2. Broker supportive services over a brief period of contact |
| Jimenez (2021) [67]  USA | Construction industry | Single interview | Members' Assistance Program (union organised) | 1. Training to set up Member's Assistance Programs  2. Access to licensed therapists/care facilities |
| LaCroix et al (2021) [49]  USA | Military  Special operations | Intervention case studies | 1. Special operations cognitive agility training (universal prevention)  2. Chaplains-CARE program in military suicide prevention (selective prevention)  3. Suicide death reviews (surveillance) | 1. Cognitive agility (the ability to deliberately adapt cognitive processing strategies in accordance with dynamic shifts in situation and environmental demands) training; 4-hour f2f small group delivery  2. Gatekeeper training for chaplains to: 1) enhance suicide intervention skills; 2) to enhance knowledge about suicide; 3) to improve beliefs and attitudes about suicide; 4) reduce reluctance to intervene; 5) to increase self-efficacy. Two versions: 1) week-long, intensive f2f training and 2) 6-hr online course.  3. Conduct suicide death reviews to generate service specific recommendations for suicide prevention. |
| Mishara and Fortin (2021) [47]  Canada | Emergency services  Police | Cohort study | Together for Life (*Ensemble pour la vie*) | See Mishara and Martin, 2012 above. |

## References

Accardi, R., Sanchez, C., Zisook, S., Hoffman, L. A. & Davidson, J. E. (2020) Sustainability and Outcomes of a Suicide Prevention Program for Nurses. *Worldviews on Evidence-Based Nursing.* 17(1)**:** 24-31.

Adamouski-Marion, K. (2020) *Evaluating the effectiveness of training used for the implementation of a peer support program to support second victims.* Doctor of Nursing Practice, Saint Francis Medical Center College of Nursuing.

Albott, C. S., Wozniak, J. R., McGlinch, B. P., Wall, M. H., Gold, B. S. & Vinogradov, S. (2020) Battle Buddies: Rapid Deployment of a Psychological Resilience Intervention for Health Care Workers During the COVID-19 Pandemic. *Anesthesia & Analgesia.* 131(1).

Arnold, J., Tango, J., Walker, I., Waranch, C., McKamie, J., Poonja, Z., & Messman, A. (2018) An evidence-based, longitudinal curriculum for resident physician wellness: The 2017 Resident Wellness Consensus Summit. *Western Journal of Emergency Medicine.* 19(2).

Baker, J. C., Bryan, C. J., Bryan, A. O. & Button, C. J. (2021) The Airman’s Edge Project: A Peer-Based, Injury Prevention Approach to Preventing Military Suicide. *International Journal of Environmental Research and Public Health* [Online], 18.

Brown, L. (2010) Reducing the suicide rate in the profession. *Veterinary Record.* 167(26)**:** 1018-1018.

Davidson, J. E., Zisook, S., Kirby, B., DeMichele, G. & Norcross, W. (2018) Suicide Prevention: A Healer Education and Referral Program for Nurses. *JONA: The Journal of Nursing Administration.* 48(2).

Doran, C. M., Ling, R., Gullestrup, J., Swannell, S. & Milner, A. (2015) The Impact of a Suicide Prevention Strategy on Reducing the Economic Cost of Suicide in the New South Wales Construction Industry. *Crisis.* 37(2)**:** 121-129.

Doran, C. M., Ling, R., Gullestrup, J., Swannell, S. & Milner, A. (2016) The Impact of a Suicide Prevention Strategy on Reducing the Economic Cost of Suicide in the New South Wales Construction Industry. *Crisis.* 37(2)**:** 121-129.

Doran, C. M., Wittenhagen, L., Heffernan, E. & Meurk, C. (2021) The MATES Case Management Model: Presenting Problems and Referral Pathways for a Novel Peer-Led Approach to Addressing Suicide in the Construction Industry. *International Journal of Environmental Research and Public Health* [Online], 18.

Downs, N., Feng, W., Kirby, B., McGuire, T., Moutier, C., Norcross, W., Norman, M., Young, I. & Zisook, S. (2014) Listening to Depression and Suicide Risk in Medical Students: the Healer Education Assessment and Referral (HEAR) Program. *Academic Psychiatry.* 38(5)**:** 547-553.

Dwyer, A. J., Morley, P., Reid, E. & Angelatos, C. (2011) Distressed doctors: a hospital-based support program for poorly performing and “at-risk” junior medical staff. *Medical Journal of Australia.* 194(9)**:** 466-469.

Ey, S., Moffit, M., Kinzie, J. M. & Brunett, P. H. (2016) Feasibility of a Comprehensive Wellness and Suicide Prevention Program: A Decade of Caring for Physicians in Training and Practice. *Journal of Graduate Medical Education.* 8(5)**:** 747-753.

Finney, E. J., Buser, S. J., Schwartz, J., Archibald, L. & Swanson, R. (2015) Suicide prevention in fire service: The Houston Fire Department (HFD) model. *Aggression and Violent Behavior.* 21**:** 1-4.

Garelick, A. I., Gross, S. R., Richardson, I., von der Tann, M., Bland, J. & Hale, R. (2007) Which doctors and with what problems contact a specialist service for doctors? A cross sectional investigation. *BMC Medicine.* 5(1)**:** 26.

Gordana, D. J. & Milivoje, P. (2007) Suicide Prevention Program in the Army of Serbia and Montenegro. *Military Medicine.* 172(5)**:** 551-555.

Gullestrup, J., Lequertier, B. & Martin, G. (2011) MATES in Construction: Impact of a Multimodal, Community-Based Program for Suicide Prevention in the Construction Industry. *International Journal of Environmental Research and Public Health* [Online], 8.

Halliwell, R. E. W. & Hoskin, B. D. (2005) Reducing the suicide rate among veterinary surgeons: how the profession can help. *Veterinary Record.* 157(14)**:** 397-398.

Jimenez, C. (2021) The Establishment of a Construction Union-Based Member Assistance Program: An Interview With Kyle Zimmer. *NEW SOLUTIONS: A Journal of Environmental and Occupational Health Policy.* 31(3)**:** 350-355.

Joseph, C., Roopa, C. G., Kumar, U., Bhatti, R. S., Panhasarathy, R., Chandramohan, V., Gupta, J. K. & Krishrnamuithy, A. (2004) Prevention of suicide: The IAM awarence training programme. *Indian Journal of Aerospace Medicine.* 48(2)**:** 8-16.

King, T. L., Batterham, P. J., Lingard, H., Gullestrup, J., Lockwood, C., Harvey, S. B., Kelly, B., LaMontagne, A. D. & Milner, A. (2019) Are Young Men Getting the Message? Age Differences in Suicide Prevention Literacy among Male Construction Workers. *International Journal of Environmental Research and Public Health* [Online], 16.

King, T. L., Gullestrup, J., Batterham, P. J., Kelly, B., Lockwood, C., Lingard, H., Harvey, S. B., LaMontagne, A. D. & Milner, A. (2018) Shifting Beliefs about Suicide: Pre-Post Evaluation of the Effectiveness of a Program for Workers in the Construction Industry. *International Journal of Environmental Research and Public Health* [Online], 15.

Knox, K. L., Litts, D. A., Talcott, G. W., Feig, J. C. & Caine, E. D. (2003) Risk of suicide and related adverse outcomes after exposure to a suicide prevention programme in the US Air Force: cohort study. *BMJ.* 327(7428)**:** 1376.

Knox, K. L., Pflanz, S., Talcott, G. W., Campise, R. L., Lavigne, J. E., Bajorska, A., Tu, X. & Caine, E. D. (2010) The US Air Force Suicide Prevention Program: Implications for Public Health Policy. *American Journal of Public Health.* 100(12)**:** 2457-2463.

Kubo, H., Urata, H., Katsuki, R., Hirashima, M., Ueno, S., Suzuki, Y., Fujisawa, D., Hashimoto, N., Kobara, K., Cho, T., Mitsui, T., Kanba, S., Otsuka, K. & Kato, T. A. (2018) Development of MHFA-based 2-h educational program for early intervention in depression among office workers: A single-arm pilot trial. *PLOS ONE.* 13(12)**:** e0208114.

LaCroix, J. M., Walsh, A., Baggett, M. A., Madison Carter, K., the Suicide Care, P., Research Initiative, T. & Ghahramanlou-Holloway, M. (2021) Three department of defense-funded public health approaches to reduce military suicide. *Suicide and Life-Threatening Behavior.* 51(2)**:** 334-343.

Lapenaite, D. & Vaicaitiene, R. (2008) Lowering suicide risk: Situation and prevention measures in the Lithuanian armed forces. *In:* Wiederhold, B. K. (ed.) *Lowering suicide risk in returning troops: Wounds of war.* Amsterdam: IOS Press.

Levenson, R. L., O'Hara, A. F. & Clark Sr, R. (2010) The Badge of Life Psychological Survival for Police Officers Program. *International Journal of Emergency Mental Health.* 12(2)**:** 95-102.

Martin, G., Swannell, S., Milner, A. & Gullestrup, J. (2016) Mates in Construction suicide prevention program: A five year review. *Journal of Community Medicine & Health Education.* 6**:** 465.

Mishara, B. L. & Fortin, L.-F. (2021) Long-Term Effects of a Comprehensive Police Suicide Prevention Program. *Crisis.* 43(3)**:** 183-189.

Mishara, B. L. & Martin, N. (2012) Effects of a Comprehensive Police Suicide Prevention Program. *Crisis.* 33(3)**:** 162-168.

Nakao, M., Nishikitani, M., Shima, S. & Yano, E. (2007) A 2-year cohort study on the impact of an Employee Assistance Programme (EAP) on depression and suicidal thoughts in male Japanese workers. *International Archives of Occupational and Environmental Health.* 81(2)**:** 151-157.

National Mental Health Commission (2017) *Review into the suicide and self-harm prevention services available to current and former serving ADF members and their families* [Online]. Australian Government. Available: https://www.dva.gov.au/sites/default/files/files/publications/health/Final_Report.pdf [Accessed 18 September 2022].

Ramchand, R., Saunders, J., Osilla, K. C., Ebener, P., Kotzias, V., Thornton, E., Strang, L. & Cahill, M. (2019) Suicide Prevention in U.S. Law Enforcement Agencies: a National Survey of Current Practices. *Journal of Police and Criminal Psychology.* 34(1)**:** 55-66.

Rø, K. E. I., Gude, T. & Aasland, O. G. (2007) Does a self-referral counselling program reach doctors in need of help? A comparison with the general Norwegian doctor workforce. *BMC Public Health.* 7(1)**:** 36.

Ross, V., Caton, N., Gullestrup, J. & Kõlves, K. (2019) Understanding the Barriers and Pathways to Male Help-Seeking and Help-Offering: A Mixed Methods Study of the Impact of the Mates in Construction Program. *International Journal of Environmental Research and Public Health* [Online], 16.

Ross, V., Caton, N., Gullestrup, J. & Kõlves, K. (2020a) A Longitudinal Assessment of Two Suicide Prevention Training Programs for the Construction Industry. *International Journal of Environmental Research and Public Health* [Online], 17.

Ross, V., Caton, N., Mathieu, S., Gullestrup, J. & Kõlves, K. (2020b) Evaluation of a Suicide Prevention Program for the Energy Sector. *International Journal of Environmental Research and Public Health* [Online], 17.

Rozanov, V. A., Mokhovikov, A. N. & Stiliha, R. (2002) Successful Model of Suicide Prevention in the Ukraine Military Environment. *Crisis.* 23(4)**:** 171-177.

Shelef, L., Laur, L., Raviv, G. & Fruchter, E. (2015) A military suicide prevention program in the Israeli Defense Force: a review of an important military medical procedure. *Disaster and Military Medicine.* 1(1)**:** 16.

Shelef, L., Tatsa-Laur, L., Derazne, E., Mann, J. J. & Fruchter, E. (2016) An effective suicide prevention program in the Israeli Defense Forces: A cohort study. *European Psychiatry.* 31**:** 37-43.

Steyn, R. (2008) The effect of multiple organizational interventions on suicidal behavior. *In: PICMET '08 - 2008 Portland International Conference on Management of Engineering & Technology, 2008*, 1924-1929.

Warner, C. H., Appenzeller, G. N., Parker, J. R., Warner, C., Diebold, C. J. & Grieger, T. (2011) Suicide Prevention in a Deployed Military Unit. *Psychiatry.* 74(2)**:** 127-141.

Wentworth, L. M. (2016) *Suicide prevention and the workplace.* Doctor of Philosophy PhD, The University of Iowa.

Willson, M. N., Robinson, J. D., McKeirnan, K. C., Akers, J. M. & Buchman, C. R. (2020) Training Student Pharmacists in Suicide Awareness and Prevention. *American Journal of Pharmaceutical Education.* 84(8)**:** ajpe847813.

Won, G. H., Lee, J. H., Choi, T. Y., Yoon, S., Kim, S. Y. & Park, J. H. (2020) The effect of a mental health promotion program on Korean firefighters. *International Journal of Social Psychiatry.* 66(7)**:** 675-681.
