# Supplementary material for: Workplace interventions to prevent suicide: A scoping review
Source: PLoS One. 2024 May 2;19(5):e0301453. doi: 10.1371/journal.pone.0301453 (PMC11065308; doi:10.1371/journal.pone.0301453)
Supplement: S2 Table — (DOCX) [file pone.0301453.s004.docx]

**Supplementary table 2. Intervention mechanisms by socioecological level**

|  |  |  |  |  | | | | | | | |  | | | | **Macrosystem** | | | | | |
| --- | --- | --- | --- | --- | --- | --- | --- | --- | --- | --- | --- | --- | --- | --- | --- | --- | --- | --- | --- | --- | --- |
|  |  |  |  | **Microsystem** | | | | | | | | **Mesosystem** | | | | **Exosystem** | | | **Chronosystem** | | |
| **Main text citation** | **Citation** | **Sector** | **Intervention** | **Education for employees [self]** | **Assessment/ screening** | **Counselling/ treatment** | **Crisis support** | **Helpline/ website** | **Health insurance** | **EAP** | **Referral/ access to external support** | **Peer support/ buddy system** | **Family involvement** | **Mentor/ supervision** | **Gatekeepers/ training** | **Policy/ procedure** | **Education for employees [others]** | **Education/ training for managers** | **Awareness campaign** | **Identify/ monitor high risk groups** | **Data/ surveillance** |
| [53] | Rozanov et al (2002) | Military | Prevention programme | x |  |  |  |  |  |  |  |  |  |  | x |  | x | x | x | x |  |
| [51] | Knox et al (2003) | Military | USAFSPP | x | x | x | x |  |  |  |  |  |  |  |  | x | x |  |  | x | x |
| [69] | Joseph et al (2004) | Military | IAM training |  |  |  |  |  |  |  |  | x |  | x |  |  | x | x |  |  |  |
| [56] | Halliwell and Hoskin (2005) | Veterinary  surgeons | Various |  | x | x |  | x |  |  |  |  |  |  |  |  |  |  |  |  |  |
| [34] | Garelick et al (2007) | Healthcare | MedNET |  |  | x |  |  |  |  |  |  |  |  |  |  |  |  |  |  |  |
| [68] | Gordana and Milivoje (2007) | Military | Suicide prevention  program |  |  |  |  |  |  |  | x | x |  |  |  |  | x |  |  |  |  |
| [25] | Nakao et al (2007) | Office  workers | EAP | x |  | x |  |  | x | x | x |  |  |  |  |  | x |  |  |  |  |
| [35] | Rø et al (2007) | Healthcare | Villa Sana |  |  | x |  |  |  |  |  |  | x |  |  |  |  |  |  |  |  |
| [54] | Lapenaite and Vaicaitiene (2008) | Military | Prevention  measures | x | x |  | x |  |  |  |  |  |  |  | x |  | x | x |  |  |  |
| [63] | Steyn (2008) | Emergency  services | Suicide prevention  workshops | x |  |  | x |  |  |  |  |  |  |  |  |  | x |  |  |  |  |
| [60] | Brown (2010) | Veterinary  surgeons | Various |  |  | x |  | x |  |  |  |  |  |  |  |  |  |  |  |  |  |
| [57] | Knox et al (2010) | Military | USAFSPP | x | x | x | x |  |  |  |  |  |  |  |  | x | x |  |  | x | x |
| [58] | Levenson et al (2010) | Emergency  services | BOL | x | x |  |  |  |  |  |  | x |  |  |  |  |  |  |  |  |  |
| [52] | Dwyer et al (2011) | Healthcare | Prevention  programme |  | x | x |  |  |  |  | x | x |  | x |  | x |  |  |  |  |  |
| [38] | Gullestrup et al (2011) | Construction  industry | MATES | x |  |  |  | x |  |  | x | x |  |  | x |  | x |  |  |  |  |
| [36] | Warner et al (2011) | Military | Suicide prevention program | x | x | x |  |  |  |  |  |  | x |  | x | x | x | x |  | x | x |
| [48] | Mishara and Martin (2012) | Emergency services | Together for Life |  | x |  |  | x |  |  |  |  |  |  |  |  | x | x | x |  |  |
| [33] | Downs et al (2014) | Healthcare | HEAR | x | x | x |  |  |  |  | x |  |  |  |  |  |  |  |  |  |  |
| [40] | Doran et al (2015) | Construction industry | MATES in  Construction | x | x |  |  | x |  |  | x | x |  |  | x |  | x |  | x |  |  |
| [64] | Finney et al (2015) | Emergency services | HFD | x |  |  |  |  |  |  |  |  |  |  |  |  | x | x | x |  |  |
| [46] | Shelef et al (2015) | Military | IDF SPP | x |  | x |  |  |  |  |  |  |  |  | x | x | x | x |  |  |  |
| [28] | Doran et al (2016) | Construction industry | MATES in Construction | x | x |  |  | x |  |  | x |  |  |  | x |  | x |  | X |  |  |
| [59] | Ey et al (2016) | Healthcare | Resident and Faculty Wellness  Program | x | x | x |  |  |  |  | x |  |  |  |  |  |  | x |  |  |  |
| [37] | Martin et al (2016) | Construction industry | MATES in Construction | x | x |  |  | x |  |  | x |  |  |  | x |  | x |  | X |  |  |
| [29] | Shelef et al (2016) | Military | IDF SPP | x |  | x |  |  |  |  |  |  |  |  | x | x | x | x |  |  |  |
| [30] | Wentworth (2016) | University | QPR training |  |  |  |  |  |  |  |  |  |  |  |  |  | x |  |  |  |  |
| [50] | National Mental Health Commission (2017) | Military | Services involved in the prevention of suicide for ADF members | x | x | x |  | x |  |  | x |  | x |  |  |  |  |  | x |  |  |
| [65] | Arnold (2018) | Healthcare | Wellness  curriculum | x |  |  |  |  |  |  |  |  |  |  |  |  |  |  |  |  |  |
| [31] | Davidson et al (2018) | Healthcare | HEAR | x | x | x |  |  |  |  | x |  |  |  |  |  |  |  |  |  |  |
| [42] | King et al (2018) | Construction  industry | GAT | x |  |  |  |  |  |  |  |  |  |  |  |  | x |  |  |  |  |
| [71] | Kubo et al (2018) | Office workers | MH first aid |  |  |  |  |  |  |  |  |  |  |  |  |  | x |  |  |  |  |
| [26] | King et al (2019) | Construction industry | GAT MATES | x |  |  |  |  |  |  |  |  |  |  |  |  | x |  |  |  |  |
| [61] | Ramchand et al (2019) | Emergency services | Current practices |  |  | x |  |  | x | x | x | x |  |  |  |  |  |  |  | x |  |
| [27] | Ross et al (2019) | Construction  industry | MATES in  Construction | x | x |  |  | x |  |  | x | x |  |  | x |  | x |  | x |  |  |
| [45] | Accardi et al (2020) | Healthcare | HEAR | x | x | x | x |  |  |  |  |  |  |  |  | x | x |  |  | x | x |
| [62] | Albott et al (2020) | Emergency  services | Battle buddies |  |  | x |  |  |  |  | x | x |  |  |  |  | x |  |  |  |  |
| [39] | Ross et al (2020a) | Construction industry | GAT | x |  |  |  |  |  |  |  |  |  |  |  |  | x |  |  |  |  |
| [39] | Ross et al (2020a) | Construction industry | MAT | x |  |  |  |  |  |  |  |  |  |  |  |  | x |  |  |  |  |
| [43] | Ross et al (2020b) | Energy  sector | GAT (MATES in  Energy) | x |  |  |  |  |  |  |  |  |  |  |  |  | x |  |  |  |  |
| [32] | Willson et al (2020) | Healthcare | Suicide Prevention for Pharmacy  Professionals |  |  |  |  |  |  |  |  |  |  |  |  |  |  |  |  |  |  |
| [55] | Won et al (2020) | Emergency services | MH promotion programme | x | x | x |  |  |  |  | x |  |  |  |  |  |  |  |  | x |  |
| [77] | Adamouski-Marion (2020) | Healthcare | CARE |  |  |  |  |  |  |  |  | x |  |  |  |  |  |  |  |  |  |
| [66] | Baker et al (2021) | Military | The Airman’s Edge Project | x |  |  |  |  |  |  |  | x |  |  |  |  |  |  |  |  |  |
| [41] | Doran et al (2021) | Construction  industry | MATES in Construction: Case management model |  | x |  |  |  |  |  | x |  |  |  | x |  |  |  |  |  |  |
| [67] | Jimenez (2021) | Construction  industry | Members' Assistance Program (union organised) |  |  |  |  |  |  | x | x |  |  |  |  |  |  |  |  |  |  |
| [49] | LaCroix et al (2021) | Military | Special operations cognitive agility training | x |  |  |  |  |  |  |  |  |  |  |  |  |  |  |  |  |  |
| [49] | LaCroix et al (2021) | Military | Chaplains-CARE program in military suicide prevention |  |  |  |  |  |  |  |  |  |  |  | x |  |  |  |  |  |  |
| [49] | LaCroix et al (2021) | Military | Suicide death  reviews |  |  |  |  |  |  |  |  |  |  |  |  |  |  |  |  |  | x |
| [47] | Mishara and Fortin (2021) | Emergency  services | Together for Life |  | x |  |  | x |  |  |  |  |  |  |  |  | x | x | x |  |  |

## References

Accardi, R., Sanchez, C., Zisook, S., Hoffman, L. A. & Davidson, J. E. (2020) Sustainability and Outcomes of a Suicide Prevention Program for Nurses. *Worldviews on Evidence-Based Nursing.* 17(1)**:** 24-31.

Adamouski-Marion, K. (2020) *Evaluating the effectiveness of training used for the implementation of a peer support program to support second victims.* Doctor of Nursing Practice, Saint Francis Medical Center College of Nursuing.

Albott, C. S., Wozniak, J. R., McGlinch, B. P., Wall, M. H., Gold, B. S. & Vinogradov, S. (2020) Battle Buddies: Rapid Deployment of a Psychological Resilience Intervention for Health Care Workers During the COVID-19 Pandemic. *Anesthesia & Analgesia.* 131(1).

Arnold, J., Tango, J., Walker, I., Waranch, C., McKamie, J., Poonja, Z., & Messman, A. (2018) An evidence-based, longitudinal curriculum for resident physician wellness: The 2017 Resident Wellness Consensus Summit. *Western Journal of Emergency Medicine.* 19(2).

Baker, J. C., Bryan, C. J., Bryan, A. O. & Button, C. J. (2021) The Airman’s Edge Project: A Peer-Based, Injury Prevention Approach to Preventing Military Suicide. *International Journal of Environmental Research and Public Health* [Online], 18.

Brown, L. (2010) Reducing the suicide rate in the profession. *Veterinary Record.* 167(26)**:** 1018-1018.

Davidson, J. E., Zisook, S., Kirby, B., DeMichele, G. & Norcross, W. (2018) Suicide Prevention: A Healer Education and Referral Program for Nurses. *JONA: The Journal of Nursing Administration.* 48(2).

Doran, C. M., Ling, R., Gullestrup, J., Swannell, S. & Milner, A. (2015) The Impact of a Suicide Prevention Strategy on Reducing the Economic Cost of Suicide in the New South Wales Construction Industry. *Crisis.* 37(2)**:** 121-129.

Doran, C. M., Ling, R., Gullestrup, J., Swannell, S. & Milner, A. (2016) The Impact of a Suicide Prevention Strategy on Reducing the Economic Cost of Suicide in the New South Wales Construction Industry. *Crisis.* 37(2)**:** 121-129.

Doran, C. M., Wittenhagen, L., Heffernan, E. & Meurk, C. (2021) The MATES Case Management Model: Presenting Problems and Referral Pathways for a Novel Peer-Led Approach to Addressing Suicide in the Construction Industry. *International Journal of Environmental Research and Public Health* [Online], 18.

Downs, N., Feng, W., Kirby, B., McGuire, T., Moutier, C., Norcross, W., Norman, M., Young, I. & Zisook, S. (2014) Listening to Depression and Suicide Risk in Medical Students: the Healer Education Assessment and Referral (HEAR) Program. *Academic Psychiatry.* 38(5)**:** 547-553.

Dwyer, A. J., Morley, P., Reid, E. & Angelatos, C. (2011) Distressed doctors: a hospital-based support program for poorly performing and “at-risk” junior medical staff. *Medical Journal of Australia.* 194(9)**:** 466-469.

Ey, S., Moffit, M., Kinzie, J. M. & Brunett, P. H. (2016) Feasibility of a Comprehensive Wellness and Suicide Prevention Program: A Decade of Caring for Physicians in Training and Practice. *Journal of Graduate Medical Education.* 8(5)**:** 747-753.

Finney, E. J., Buser, S. J., Schwartz, J., Archibald, L. & Swanson, R. (2015) Suicide prevention in fire service: The Houston Fire Department (HFD) model. *Aggression and Violent Behavior.* 21**:** 1-4.

Garelick, A. I., Gross, S. R., Richardson, I., von der Tann, M., Bland, J. & Hale, R. (2007) Which doctors and with what problems contact a specialist service for doctors? A cross sectional investigation. *BMC Medicine.* 5(1)**:** 26.

Gordana, D. J. & Milivoje, P. (2007) Suicide Prevention Program in the Army of Serbia and Montenegro. *Military Medicine.* 172(5)**:** 551-555.

Gullestrup, J., Lequertier, B. & Martin, G. (2011) MATES in Construction: Impact of a Multimodal, Community-Based Program for Suicide Prevention in the Construction Industry. *International Journal of Environmental Research and Public Health* [Online], 8.

Halliwell, R. E. W. & Hoskin, B. D. (2005) Reducing the suicide rate among veterinary surgeons: how the profession can help. *Veterinary Record.* 157(14)**:** 397-398.

Jimenez, C. (2021) The Establishment of a Construction Union-Based Member Assistance Program: An Interview With Kyle Zimmer. *NEW SOLUTIONS: A Journal of Environmental and Occupational Health Policy.* 31(3)**:** 350-355.

Joseph, C., Roopa, C. G., Kumar, U., Bhatti, R. S., Panhasarathy, R., Chandramohan, V., Gupta, J. K. & Krishrnamuithy, A. (2004) Prevention of suicide: The IAM awarence training programme. *Indian Journal of Aerospace Medicine.* 48(2)**:** 8-16.

King, T. L., Batterham, P. J., Lingard, H., Gullestrup, J., Lockwood, C., Harvey, S. B., Kelly, B., LaMontagne, A. D. & Milner, A. (2019) Are Young Men Getting the Message? Age Differences in Suicide Prevention Literacy among Male Construction Workers. *International Journal of Environmental Research and Public Health* [Online], 16.

King, T. L., Gullestrup, J., Batterham, P. J., Kelly, B., Lockwood, C., Lingard, H., Harvey, S. B., LaMontagne, A. D. & Milner, A. (2018) Shifting Beliefs about Suicide: Pre-Post Evaluation of the Effectiveness of a Program for Workers in the Construction Industry. *International Journal of Environmental Research and Public Health* [Online], 15.

Knox, K. L., Litts, D. A., Talcott, G. W., Feig, J. C. & Caine, E. D. (2003) Risk of suicide and related adverse outcomes after exposure to a suicide prevention programme in the US Air Force: cohort study. *BMJ.* 327(7428)**:** 1376.

Knox, K. L., Pflanz, S., Talcott, G. W., Campise, R. L., Lavigne, J. E., Bajorska, A., Tu, X. & Caine, E. D. (2010) The US Air Force Suicide Prevention Program: Implications for Public Health Policy. *American Journal of Public Health.* 100(12)**:** 2457-2463.

Kubo, H., Urata, H., Katsuki, R., Hirashima, M., Ueno, S., Suzuki, Y., Fujisawa, D., Hashimoto, N., Kobara, K., Cho, T., Mitsui, T., Kanba, S., Otsuka, K. & Kato, T. A. (2018) Development of MHFA-based 2-h educational program for early intervention in depression among office workers: A single-arm pilot trial. *PLOS ONE.* 13(12)**:** e0208114.

LaCroix, J. M., Walsh, A., Baggett, M. A., Madison Carter, K., the Suicide Care, P., Research Initiative, T. & Ghahramanlou-Holloway, M. (2021) Three department of defense-funded public health approaches to reduce military suicide. *Suicide and Life-Threatening Behavior.* 51(2)**:** 334-343.

Lapenaite, D. & Vaicaitiene, R. (2008) Lowering suicide risk: Situation and prevention measures in the Lithuanian armed forces. *In:* Wiederhold, B. K. (ed.) *Lowering suicide risk in returning troops: Wounds of war.* Amsterdam: IOS Press.

Levenson, R. L., O'Hara, A. F. & Clark Sr, R. (2010) The Badge of Life Psychological Survival for Police Officers Program. *International Journal of Emergency Mental Health.* 12(2)**:** 95-102.

Martin, G., Swannell, S., Milner, A. & Gullestrup, J. (2016) Mates in Construction suicide prevention program: A five year review. *Journal of Community Medicine & Health Education.* 6**:** 465.

Mishara, B. L. & Fortin, L.-F. (2021) Long-Term Effects of a Comprehensive Police Suicide Prevention Program. *Crisis.* 43(3)**:** 183-189.

Mishara, B. L. & Martin, N. (2012) Effects of a Comprehensive Police Suicide Prevention Program. *Crisis.* 33(3)**:** 162-168.

Nakao, M., Nishikitani, M., Shima, S. & Yano, E. (2007) A 2-year cohort study on the impact of an Employee Assistance Programme (EAP) on depression and suicidal thoughts in male Japanese workers. *International Archives of Occupational and Environmental Health.* 81(2)**:** 151-157.

National Mental Health Commission (2017) *Review into the suicide and self-harm prevention services available to current and former serving ADF members and their families* [Online]. Australian Government. Available: https://www.dva.gov.au/sites/default/files/files/publications/health/Final_Report.pdf [Accessed 18 September 2022].

Ramchand, R., Saunders, J., Osilla, K. C., Ebener, P., Kotzias, V., Thornton, E., Strang, L. & Cahill, M. (2019) Suicide Prevention in U.S. Law Enforcement Agencies: a National Survey of Current Practices. *Journal of Police and Criminal Psychology.* 34(1)**:** 55-66.

Rø, K. E. I., Gude, T. & Aasland, O. G. (2007) Does a self-referral counselling program reach doctors in need of help? A comparison with the general Norwegian doctor workforce. *BMC Public Health.* 7(1)**:** 36.

Ross, V., Caton, N., Gullestrup, J. & Kõlves, K. (2019) Understanding the Barriers and Pathways to Male Help-Seeking and Help-Offering: A Mixed Methods Study of the Impact of the Mates in Construction Program. *International Journal of Environmental Research and Public Health* [Online], 16.

Ross, V., Caton, N., Gullestrup, J. & Kõlves, K. (2020a) A Longitudinal Assessment of Two Suicide Prevention Training Programs for the Construction Industry. *International Journal of Environmental Research and Public Health* [Online], 17.

Ross, V., Caton, N., Mathieu, S., Gullestrup, J. & Kõlves, K. (2020b) Evaluation of a Suicide Prevention Program for the Energy Sector. *International Journal of Environmental Research and Public Health* [Online], 17.

Rozanov, V. A., Mokhovikov, A. N. & Stiliha, R. (2002) Successful Model of Suicide Prevention in the Ukraine Military Environment. *Crisis.* 23(4)**:** 171-177.

Shelef, L., Laur, L., Raviv, G. & Fruchter, E. (2015) A military suicide prevention program in the Israeli Defense Force: a review of an important military medical procedure. *Disaster and Military Medicine.* 1(1)**:** 16.

Shelef, L., Tatsa-Laur, L., Derazne, E., Mann, J. J. & Fruchter, E. (2016) An effective suicide prevention program in the Israeli Defense Forces: A cohort study. *European Psychiatry.* 31**:** 37-43.

Steyn, R. (2008) The effect of multiple organizational interventions on suicidal behavior. *In: PICMET '08 - 2008 Portland International Conference on Management of Engineering & Technology, 2008*, 1924-1929.

Warner, C. H., Appenzeller, G. N., Parker, J. R., Warner, C., Diebold, C. J. & Grieger, T. (2011) Suicide Prevention in a Deployed Military Unit. *Psychiatry.* 74(2)**:** 127-141.

Wentworth, L. M. (2016) *Suicide prevention and the workplace.* Doctor of Philosophy PhD, The University of Iowa.

Willson, M. N., Robinson, J. D., McKeirnan, K. C., Akers, J. M. & Buchman, C. R. (2020) Training Student Pharmacists in Suicide Awareness and Prevention. *American Journal of Pharmaceutical Education.* 84(8)**:** ajpe847813.

Won, G. H., Lee, J. H., Choi, T. Y., Yoon, S., Kim, S. Y. & Park, J. H. (2020) The effect of a mental health promotion program on Korean firefighters. *International Journal of Social Psychiatry.* 66(7)**:** 675-681.
