# Supplementary material for: Workplace interventions to prevent suicide: A scoping review
Source: PLoS One. 2024 May 2;19(5):e0301453. doi: 10.1371/journal.pone.0301453 (PMC11065308; doi:10.1371/journal.pone.0301453)
Supplement: Attachment — Submitted filename: PONE-D-23-07980_reviewer.pdf [file pone.0301453.s005.pdf]

PLOS ONE

Organisational suicide prevention interventions: A scoping review

--Manuscript Draft--

**Manuscript Number:**	PONE-D-23-07980
**Article Type:**	Research Article
**Full Title:**	Organisational suicide prevention interventions: A scoping review
**Short Title:**	Organisational suicide prevention interventions
**Corresponding Author:**	Nutmeg Hallett<br>University of Birmingham<br>Birmingham, UNITED KINGDOM
**Keywords:**	Suicide prevention; workplace; realist methods; scoping review
**Abstract:**	OBJECTIVES: To examine organisational interventions for workplace suicide prevention, identifying the effects, mechanisms, moderators, implementation and economic costs, and how interventions are evaluated.<br>BACKGROUND: Suicide is a devastating event that can have a profound and lasting impact on the individuals and families affected, with the highest rates found among adults of work age. Employers have a legal and ethical responsibility to provide a safe working environment for their employees, which includes addressing the issue of suicide and promoting mental health and well-being.<br>METHODS: A realist perspective was taken, to identify within organisational suicide prevention interventions, what works, for whom and in what circumstances. Published and unpublished studies in six databases were searched. To extract and map data on the interventions the Effect, Mechanism, Moderator, Implementation, Economic (EMMIE) framework was used. Mechanisms were deductively analysed against Bronfenbrenner's socio-ecological model.<br>RESULTS: From 3187 records screened, 46 papers describing 36 interventions within the military, healthcare, the construction industry, emergency services, office workers, veterinary surgeons, the energy sector and higher education. Most mechanisms were aimed at the individual's immediate environment, with the most common being education or training on recognising signs of stress, suicidality or mental illness in oneself. Studies examined the effectiveness of interventions in terms of suicide rates, suicidality or symptoms of mental illness, and changes in perceptions, attitudes or beliefs, with most reporting positive results. Few studies reported economic costs but those that did suggested that the interventions are cost-effective.<br>CONCLUSIONS: It seems likely that organisational suicide prevention programmes can have a positive impact on attitudes and beliefs towards suicide as well reducing the risk of suicide. Education, to support individuals to recognise the signs and symptoms of stress, mental ill health and suicidality in both themselves and others, is likely to be an effective starting point for successful interventions.
**Order of Authors:**	Nutmeg Hallett
	Helen Rees, MSc, BNurs (Hons)
	Felicity Hannah
	Lorna Hollowood
	Caroline Bradbury-Jones
**Additional Information:**	
**Question**	**Response**
**Financial Disclosure**<br><br>Enter a financial disclosure statement that describes the sources of funding for the work included in this submission. Review the submission guidelines for detailed	NH and HR received funding from NHS England to conduct this review. The funders had no role in the study design, data collection and analysis, decision to publish, or preparation of the manuscript.

requirements. View published research articles from *PLOS ONE* for specific examples.<br><br>This statement is required for submission and **will appear in the published article** if the submission is accepted. Please make sure it is accurate.<br><br>**Unfunded studies**<br>Enter: *The author(s) received no specific funding for this work.*<br><br>**Funded studies**<br>Enter a statement with the following details:<br>• Initials of the authors who received each award<br>• Grant numbers awarded to each author<br>• The full name of each funder<br>• URL of each funder website<br>• Did the sponsors or funders play any role in the study design, data collection and analysis, decision to publish, or preparation of the manuscript?<br>• **NO** - Include this sentence at the end of your statement: *The funders had no role in study design, data collection and analysis, decision to publish, or preparation of the manuscript.*<br>• **YES** - Specify the role(s) played.<br><br>* typeset	
**Competing Interests**<br><br>Use the instructions below to enter a competing interest statement for this submission. On behalf of all authors, disclose any competing interests that could be perceived to bias this work—acknowledging all financial support and any other relevant financial or non-financial competing interests.<br><br>This statement is required for submission and **will appear in the published article** if the submission is accepted. Please make sure it is accurate and that any funding sources listed in your Funding Information later in the submission form are also declared in your Financial Disclosure	The authors have declared that no competing interests exist.

statement.<br><br>View published research articles from *PLOS ONE* for specific examples.<br><br>**NO authors have competing interests**<br><br>Enter: *The authors have declared that no competing interests exist.*<br><br>**Authors with competing interests**<br><br>Enter competing interest details beginning with this statement:<br><br>*I have read the journal's policy and the authors of this manuscript have the following competing interests: [insert competing interests here]*<br><br>* typeset	
**Ethics Statement**<br><br>Enter an ethics statement for this submission. This statement is required if the study involved:<br><br>• Human participants<br>• Human specimens or tissue<br>• Vertebrate animals or cephalopods<br>• Vertebrate embryos or tissues<br>• Field research<br><br>Write "N/A" if the submission does not require an ethics statement.<br><br>General guidance is provided below. Consult the submission guidelines for detailed instructions. **Make sure that all information entered here is included in the Methods section of the manuscript.**	N/A

**Format for specific study types**<br><br>**Human Subject Research (involving human participants and/or tissue)**<br>• Give the name of the institutional review board or ethics committee that approved the study<br>• Include the approval number and/or a statement indicating approval of this research<br>• Indicate the form of consent obtained (written/oral) or the reason that consent was not obtained (e.g. the data were analyzed anonymously)<br><br>**Animal Research (involving vertebrate animals, embryos or tissues)**<br>• Provide the name of the Institutional Animal Care and Use Committee (IACUC) or other relevant ethics board that reviewed the study protocol, and indicate whether they approved this research or granted a formal waiver of ethical approval<br>• Include an approval number if one was obtained<br>• If the study involved *non-human primates*, add *additional details* about animal welfare and steps taken to ameliorate suffering<br>• If anesthesia, euthanasia, or any kind of animal sacrifice is part of the study, include briefly which substances and/or methods were applied<br><br>**Field Research**<br><br>Include the following details if this study involves the collection of plant, animal, or other materials from a natural setting:<br>• Field permit number<br>• Name of the institution or relevant body that granted permission	
**Data Availability**<br><br>Authors are required to make all data underlying the findings described fully available, without restriction, and from the time of publication. PLOS allows rare exceptions to address legal and ethical concerns. See the PLOS Data Policy and FAQ for detailed information.	Yes - all data are fully available without restriction

A Data Availability Statement describing where the data can be found is required at submission. Your answers to this question constitute the Data Availability Statement and **will be published in the article**, if accepted.<br><br>**Important:** Stating 'data available on request from the author' is not sufficient. If your data are only available upon request, select 'No' for the first question and explain your exceptional situation in the text box.<br><br>Do the authors confirm that all data underlying the findings described in their manuscript are fully available without restriction?	
**Describe where the data may be found in full sentences. If you are copying our sample text, replace any instances of XXX with the appropriate details.**<br><br>• If the data are **held or will be held in a public repository**, include URLs, accession numbers or DOIs. If this information will only be available after acceptance, indicate this by ticking the box below. For example: *All XXX files are available from the XXX database (accession number(s) XXX, XXX.).*<br>• If the data are all contained **within the manuscript and/or Supporting Information files**, enter the following: *All relevant data are within the manuscript and its Supporting Information files.*<br>• If neither of these applies but you are able to provide **details of access elsewhere**, with or without limitations, please do so. For example:<br><br>*Data cannot be shared publicly because of [XXX]. Data are available from the XXX Institutional Data Access / Ethics Committee (contact via XXX) for researchers who meet the criteria for access to confidential data.*<br><br>*The data underlying the results presented in the study are available from (include the name of the third party*	All relevant data are within the manuscript and its Supporting Information files.

and contact information or URL).<br>• This text is appropriate if the data are owned by a third party and authors do not have permission to share the data.<br><br>* typeset	
Additional data availability information:	

Powered by Editorial Manager® and ProduXion Manager® from Aries Systems Corporation

Manuscript

Click here to access/download;Manuscript;Organisational suicide prevention scoping review_Manuscript.docx

Organisational suicide prevention interventions: A scoping review

Nutmeg Hallett[1*]

Helen Rees[2]

Felicity Hannah[1]

Lorna Hollowood[3]

Caroline Bradbury-Jones[1]

[1]School of Nursing and Midwifery, University of Birmingham, Birmingham, United Kingdom

[2]Health and Allied Professionals, Nottingham Trent University, NG11 8NS

[3]Three Counties School of Nursing and Midwifery, University of Worcester, WR2 6AJ

* Corresponding author

E-mail: n.n.hallett@bham.ac.uk

Abstract

OBJECTIVES: To examine organisational interventions for workplace suicide prevention, identifying the effects, mechanisms, moderators, implementation and economic costs, and how interventions are evaluated.

BACKGROUND: Suicide is a devastating event that can have a profound and lasting impact on the individuals and families affected, with the highest rates found among adults of work age. Employers have a legal and ethical responsibility to provide a safe working environment for their employees, which includes addressing the issue of suicide and promoting mental health and well-being.

METHODS: A realist perspective was taken, to identify within organisational suicide prevention interventions, what works, for whom and in what circumstances. Published and unpublished studies in six databases were searched. To extract and map data on the interventions the Effect, Mechanism, Moderator, Implementation, Economic (EMMIE) framework was used. Mechanisms were deductively analysed against Bronfenbrenner's socio-ecological model.

RESULTS: From 3187 records screened, 46 papers describing 36 interventions within the military, healthcare, the construction industry, emergency services, office workers, veterinary surgeons, the energy sector and higher education. Most mechanisms were aimed at the individual's immediate environment, with the most common being education or training on recognising signs of stress, suicidality or mental illness in oneself. Studies examined the effectiveness of interventions in terms of suicide rates, suicidality or symptoms of mental illness, and changes in perceptions, attitudes or beliefs, with most reporting positive results. Few studies reported economic costs but those that did suggested that the interventions are cost-effective.

CONCLUSIONS: It seems likely that organisational suicide prevention programmes can have a positive impact on attitudes and beliefs towards suicide as well reducing the risk of suicide. Education, to support individuals to recognise the signs and symptoms of stress, mental ill health and suicidality in both themselves and others, is likely to be an effective starting point for successful interventions.

Introduction

Annually, more than 700,000 die by suicide, with many more making a suicide attempt [1]. Rates of suicide are highest in people of working age; in England suicide is one of the leading causes of death in those aged 20-64 [2]. Suicide is a devastating event that can have a profound and lasting impact on the individuals and families affected, including increasing their risk of suicide [3].

Suicide is a multifaceted phenomenon, which creates challenges to creating prevention strategies due to the complex convergence of risk factors from genetic to psychosocial to cultural [4]. Whilst employment is a protective factor, specific aspects including job pressure and high stress can contribute to increased suicide risk in some occupational groups. Barriers for the working population in help-seeking behaviour include concerns regarding potential career impact, stigma and confidentiality [5]. Most people who die by suicide do not have formal contact with mental health services (The National Confidential Inquiry into Suicide and Safety in Mental Health (NCISH), 2020). This means that organisations can play a key role in suicide prevention.

Employers have a legal and ethical responsibility to provide a safe working environment for their employees [6]. This includes addressing the issue of suicide and promoting mental health and well-being. Moreover, the loss of an employee to suicide can have a profound impact on the morale and productivity of the remaining staff, leading to increased absenteeism, decreased motivation, and decreased productivity [7]. Therefore, preventing suicide is important for organizations to support the well-being of their employees, fulfil their legal and ethical responsibilities, and maintain a healthy and productive workplace.

Despite the importance of suicide prevention as an organisational activity, the research evidence underpinning such activities is limited. Workplace psychosocial interventions that are aimed at preventing and treating anxiety and depression, particularly those based on cognitive-behavioural therapy, are effective in reducing symptoms [8, 9]. As some psychological symptoms, such as low self-esteem, hopelessness and helplessness, as well as major depressive disorder, are risk factors for suicide [10], it is likely that such interventions could play a role in suicide prevention. Suicide risk factors are, however, wider ranging than psychological symptoms, and therefore suicide prevention interventions may need to include more than therapy. The literature on such interventions is growing. In 2008, Takada and Shima [11] identified 16 workplace programmes, and that they included education and training for individuals and managers, developing support networks and cooperation between internal and external resources. In 2017, Witt and colleagues [12] identified 13 studies that focused specifically on interventions aimed at emergency and protective services employees. They found that such interventions reduced suicide rates but identified that further research was needed. To the best of our knowledge, there are no reviews that have examined the contexts by which workplace suicide prevention interventions produce an effect.

The aim of this review was to examine organisational interventions for workplace suicide prevention. We sought to i) identify the effects, mechanisms, moderators, implementation and economic costs of suicide prevention interventions, ii) identify how workplace suicide prevention interventions are evaluated and iii) make recommendations for practice and research.

Methods

Protocol and registration

The protocol for this review was developed by NH, HR, LH and CBJ and registered with the Open Science Framework (OSF; doi: 10.17605/OSF.IO/9GFRV). The review was conducted in accordance with guidance from the Joanna Briggs Institute [13] and is reported in accordance with the PRISMA statement for reporting scoping reviews [14].

Conceptual model

The complexity of the workplace suicide prevention interventions means that evaluation lends itself to the realist perspective of identifying what works, for whom and in what circumstances, rather than traditional scientific approaches aimed at identifying effect size within a specified confidence interval [15]. Realism seeks to explain how an intervention works in terms of the interaction between the context, mechanism and outcomes [16], where context refers to the situation around the person, mechanisms are the causal forces that allow an understanding of the relationship between the context and the outcome, and outcomes can be intended and unintended [17].

Data sources and search strategy

Published and unpublished studies in CINAHL Plus, EMBASE, Ovid Medline, ProQuest including Theses and Dissertations, and Web of Science Core Collection were searched using key terms related to i) suicide and suicide prevention, ii) workplace and iii) intervention. Subject headings were searched where available and searches were combined with Boolean operators, see Table 1. A supplementary search was conducted in Google Scholar with results being extracted using Harzing's Publish or Perish software. Studies were limited to those published in English between January 2002 and August 2022 when the database searching was conducted. Results were uploaded to Rayyan for de-duplication and screening.

Table 1. Search terms by database

Database	Search terms	No. results
CINAHL Plus	To be addec	248
EMBASE	(suicide [MH] OR suicide OR "suicide prevention") AND (workplace [MH] OR workplace OR organi?ation OR workforce [MH] OR workforce) AND (intervention OR program? OR response OR strateg?)	1063
Google Scholar	(suicide OR "suicide prevention") AND (workplace OR organisation OR organization OR workforce) AND (intervention OR program OR programme OR strategy OR strategies)	980
Ovid MEDLINE	(suicide [MH] OR suicide OR "suicide prevention") AND (workplace [MH] OR workplace OR organi?ation OR workforce [MH] OR workforce) AND (intervention OR program? OR response OR strateg?)	742
PsycINFO	(suicide [MH] OR suicide OR "suicide prevention") AND (workplace [MH] OR workplace OR organi?ation OR workforce [MH] OR workforce) AND (intervention OR program? OR response OR strateg?)	387
ProQuest	(noft(suicide OR "suicide prevention")) AND (noft(workplace OR organisation OR organization OR workforce)) AND (noft((intervention OR program OR programme OR strategy OR strategies))	500
Web of Science Core Collection	TS=(suicide OR "suicide prevention") AND TS=(workplace OR organisation OR organization OR workforce) AND TS=(intervention OR program OR programme OR strategy OR strategies)	918

Eligibility criteria

Eligibility criteria were based on population, concept, context and types of evidence, see Table 2.

Table 2. Eligibility criteria for inclusion in scoping review

	Inclusion criteria
Population	Workforce
Concept	Suicide prevention interventions or programmes
Context	Organisations/workplaces
Types of evidence	Any primary research; quality improvement; audit. Systematic reviews were used to identify primary research meeting the inclusion criteria.

Screening and selection process

At least two reviewers screened each result by title and abstract, with disagreements resolved by discussion with the review team. The remaining full text papers were accessed and screened against the eligibility criteria, again by at least two reviewers with disagreements resolved by discussion.

Data charting, extraction and synthesis

Study characteristics were extracted as defined in the protocol. To extract and map data on the interventions the Effect, Mechanism, Moderator, Implementation, Economic (EMMIE) framework was used [18], see Table 3. Not only does this work well for highlighting evidence gaps, but it also assists with exploring suicide prevention interventions in terms of what works, for who and in what circumstances, in accordance with realist methods.

Table 3. Operationalisation of EMMIE (adapted from Johnson et al., 2015)

	Description	Data to be extracted
Effect	Overall effect size and direction of effect	Outcome(s) studied, effect size and direction
Mechanisms	How the intervention produces its effects	Breakdown of elements of interventions
Moderators	Contexts that moderate if mechanisms will be activated to generate the intended effect	Contextual conditions for the intervention
Implementation	Barriers and facilitators of the intervention	Documented barriers/facilitators
Economic	Is the intervention cost-effective; cost-benefit analysis	Any analysis of costs

Synthesis of the mechanisms of the interventions was conducted in a two-stage process to allow us to synthesise the interventions by type. First, mechanisms were thematically analysed by a process of coding and inductively grouping the mechanisms into themes. Second, deductive analysis was conducted to apply data to Bronfenbrenner’s [19] socio-ecological model. Bronfenbrenner’s ecological model is a popular theory in the social sciences because it offers a framework through which to examine individuals' relationships within their immediate context, their communities and wider society. In the context of this review, an ecological approach conceptualises suicide as a multifaceted phenomenon, grounded in interplay among personal, situational and socio-cultural factors. We used it to organise the mechanisms of interventions in the results section. This model enabled us to determine the interventions that target suicide prevention at different levels and allows us to consider the interplay between individual factors, and social and environmental

influences. Moderators, implementation and economic costs were narratively synthesised by grouping data within and between studies and exploring relationships [20].

Quality assessment

In accordance with the JBI guidance, no assessment of the quality of the included studies was undertaken [13].

Results

Search results

From 3187 records screened, 46 papers describing 36 interventions were included in this scoping review, see Fig 1 [21].

Fig 1. about here

Characteristics of included studies

Of the included studies, 17 were published between 2002 and 2012 and the remaining 30 were published in the last 10 years, details provided in Supplementary Table 1. Geographically the studies were conducted across the globe with 21 in North America, 12 in Australasia, 7 in Europe, 6 in Asia and 1 in Africa. Three studies were conducted in Low- and Middle-income Countries (LMIC; India, South Africa, Ukraine). A variety of sectors were represented: military (*k*=12), healthcare (*k*=11), the construction industry (*k*=10), emergency services (ambulance, fire, police; *k*=8), office workers (*k*=2), veterinary surgeons (*k*=2) and one each in the energy sector and a university.

A minority of studies (*k*=16) provided data on gender and four studies only included males [22-25]. All but two of the non-healthcare-based studies that described the gender ratio of participants (*k*=6) included a high male to female participant ratio (73.2%-92.1% male). One military-based study had an almost even split by gender, with 52.4% males [26] and the university-based intervention included 85.6% female participants [27]. Of the 11 interventions aimed at healthcare workers, five did not provide data on gender, and of those that did (*k*=6) ratios were mixed. More female nurses engaged with HEAR [28]. There were more female students in the pharmacy [29] and medical [30] cohorts. An equal gender mix of medical doctors presented to services aimed at doctors [31, 32]. Two studies included suicide rates by gender; in one military study, four out of five suicides were completed by men [33] and in the construction industry all but two of 426 suicides were completed by men [34].

Interventions

The 47 included studies described 36 different interventions; details provided in Supplementary Table 2. Two interventions were explored across multiple studies. Nine studies examined MATES in Construction, a multi-modal programme aimed at Australian construction workers, either as a whole programme [24, 25, 34-37] or in part [23, 38, 39]. One study described a spin-off from this programme, MATES in Energy, aimed at workers in the energy industry [40]. The programme involves general awareness training and training workers to improve mental health and suicide prevention literacy, with the aim of increasing help-seeking behaviours; recruiting volunteer 'connectors' to act as gatekeepers; key-worker suicide first aid 'Applied Suicide Intervention Skills Training' (ASIST); MATES field officers to support workplace volunteers; a support line; and case management for workers at risk of suicide [41]. The Healer Education Assessment and Referral (HEAR) programme was developed to increase mental health service utilisation and reduce suicide

risk among medical school students [30] and was subsequently adapted for use with nurses [28, 42]. HEAR has a two-pronged approach: i) educational training on depression and suicide with the aim of destigmatising mental health treatment and ii) web-based screening, and assessment and referral for those identified at risk [43].

Of the other interventions two were described in initial and follow-up studies: i) the Israeli Defence Force Suicide Prevention Program [44, 45] and ii) Together for Life, a suicide prevention programme for the Montreal police [46, 47]. One study described three separate interventions [48] and the remaining interventions were described in single studies only.

Mechanisms of interventions by socio-ecological level

Most mechanisms were aimed at the microsystem, that is, an individual's immediate environment, see Table 4, and this was the case across sectors. The most common mechanism at this level was education or training on recognising signs of stress, suicidality or mental illness in oneself, an element of 18 of the 37 interventions. This was followed by counselling or treatment (n=15), and 12 interventions each including assessment or screening and/or referral to external support. The other mechanisms at this level were only seen in 2-5 interventions each.

At the mesosystem level the most common mechanisms were providing a peer support or buddy system (n=9) and gatekeeper training (n=6). Only three interventions included family members; the National Mental Health Commission [49] described the interventions available for Australian Defence Force (ADF) members. Family was included in many of the interventions, for example involving family members in supporting service personnel when leaving the ADF, and services aimed specifically at families such as an advocacy service and helpline. Similarly, workshops were delivered for the families and spouses of soldiers transitioning to home after service in the US military [50]. This intervention also included in-service training for spouses aimed at increase soldier resilience. The Villa Sana programme offered one-day or week-long counselling for doctors individually or with their spouse or partner [51].

Training was the most common feature of interventions aimed at the exosystem, i.e. the indirect environment. Eighteen interventions included training for employees or managers aimed at recognising signs of stress, suicidality or mental illness in others. Only three studies described changes to policy, of which two were in the military and one was in healthcare. The Israeli Defence Force suicide prevention programme included a weapon availability reduction policy, ordering soldiers to keep their personal weapons in locked storage when on leave, to reduce availability of means of suicide [52]. The US Air Force introduced an investigative interview policy to ensure that individuals under investigation for legal problems are assessed for risk of suicide [52]. A general hospital in Australia introduced open-door policies for the medical education and medical workforce units to allow for informal two-way communication between junior doctors and management, with the aim of creating a supportive work culture [53].

Awareness campaigns, the only intervention at the macrosystem level, were a feature of four interventions, of which two were aimed at the military. The Ukraine military developed booklets for all soldiers, containing information about suicide and what a soldier can do if they identify another soldier at risk [54]. The Australian Defence Force uses online information resources and e-health services to raise awareness and improve mental health literacy [55]. One of the key facets of MATES in Construction is raising awareness of suicide and mental health through general awareness training, offered to all workers [56]. A publicity campaign aimed at the Montreal police force included articles in police newspapers, posters hung in police units and brochures distributed to all members of the force [57].

Table 4. Mechanisms of interventions by Socio-Ecological Model (SEM) level

SEM level	Mechanism	Description
Microsystem (Immediate Environment)	Assessment/screening	Assessment or screening within the intervention for mental health and/or suicide risks.
	Counselling/treatment	Provision of counselling or treatment (medication) within the intervention for mental health and/or suicide risk.
	Crisis support	Providing support within the intervention for people at immediate risk of suicide.
	Education for employees (self)	Education or training provided on recognition/management of stress/mental illness/suicidality in oneself.
	Employee assistant programme (EAP)	Employee benefit programmes aimed at helping employees deal with personal problems/issues that may affect their performance, health and wellbeing.
	Health insurance	Provision of employee health insurance for mental health.
	Helpline/website	Provision of a helpline or website for people to access support with mental health/suicidality.
	Referral/access to external support	Providing referral or access to external support e.g. for counselling/treatment.
Mesosystem (Connections)	Family involvement	Providing support to families or enabling family members to be included in the intervention.
	Gatekeepers	Training or identification of people, from within and without of the organisation, who are strategically to recognise and refer someone at risk of suicide.
	Mentor/ supervision	Provision of either mentorship or supervision from managers/superiors to provide support with, or identify signs/risks of, stress/mental illness/suicidality.
	Peer support/ buddy system	Training or identification of peers to provide support with, or identify signs/risks of, stress/mental illness/suicidality.
Exosystem (Indirect Environment)	Education/ training for employees [others] or managers	Education or training provided on recognition/management of stress/mental illness/suicidality in others.
	Policy	The introduction/adaption of organisation-wide policies explicitly described in relation to suicide prevention.
Macrosystem (Social and Cultural Values)	Awareness campaign	Raising awareness of suicidality through leaflets/posters etc.
Chronosystem (Changes Over Time)	Data surveillance	Collection of data on suicides, risk profiles etc.
	Identifying/ monitoring high risk groups	Using data to identify high-risk groups within the workforce, or time points when risks are increased (e.g. on discharge from the military).

Effectiveness of interventions

Interventions were evaluated in fewer than half the studies in this review. In total, 20 studies examined the effectiveness of interventions in terms of suicide rates (n=10), suicidality or symptoms of mental illness (n=2), and changes in perceptions, attitudes or beliefs (n=8). Of the 10 studies that examined suicide rates 9 found a decrease in suicide rates post-intervention implementation, and of these, 7 reported a statistically significant decrease. After implementation of Together for Life in Montreal, there was a 78.9% decrease in suicide rates among Montreal police compared with a non-significant increase for police in Quebec acting as the control [58] and rates remained significantly lower in Montreal when compared with Quebec in the subsequent 10 years [59]. Similarly a

significant decrease in rates of suicide was reported in the Serbia and Montenegro [44], Ukrainian [44] and US [59] military post-intervention. Three studies provided risk calculations. An evaluation of MATES found a 9.64% decrease in suicide risk [44]. There was a 33% relative risk reduction in the exposed cohort of the US Air Force [48] and a relative risk of 0.52 post implementation in the Israeli military [52]. After implementation of IAM awareness training there were no suicides in the follow-up period in the Indian Air Force, however the authors note that rates of suicide were very low prior to implementation [60]. Amongst South African police there was no correlation between the number of suicide prevention workshops delivered and the number of officer suicides by region [52].

Two studies found a reduction in depression symptoms and suicidal ideation following intervention implementation, both conducted in Asia [44, 46], although there was no significant reduction of depression as assessed by the Hamilton Depression Scale [22]. Of these, one also found reductions in agitation and guilt [22] and the other in anxiety, insomnia and alcohol use [61].

Improvements in confidence in the application of skills or intervening with a colleague at risk of suicide after training were seen in three studies across industries [24, 29, 62] whilst increased help-seeking intentions were reported by two evaluations of MATES [24, 36]. Shifts towards more favourable attitudes and beliefs about suicide were also reported after MATES with the youngest respondents demonstrating the greatest intervention-associated change [23, 39]. Other changes in perceptions were related to improved suicide-literacy [36, 39] and perceptions of the safety climate [27].

Moderators and implementation barriers/facilitators

Few studies identified moderators (*k*=18), i.e. the contexts that moderate whether a mechanism will be activated to generate an effect, or implementation barriers/facilitators (*k*=6). There were two main moderators identified. The first was that the intervention should be tailored to the specific context i.e. the police [63] or the construction industry [25, 36, 37], or that there was an in-house suicide prevention team [64]. Secondly, and perhaps more importantly, was the guarantee that information about suicide assessment or treatment would not appear in personnel records. This was a key moderator for interventions in the military [65], the police [66], and aimed at medics [66] and emergency healthcare workers [67].

Lack of time was identified as a barrier to implementation [68] whilst providing dedicated time was a facilitator [64]. Other barriers were a lack of information about the programme [68], intervention implementation diminishing over time [65] and difficulties in earning the trust of workers [69]. Facilitators of the MATES in Construction programme were promoting the programme on site [24] and being easy to engage with [38].

Economic costs

Only four studies provided detail about economic costs, two relating to MATES and the others in healthcare. The MATES evaluations use the same figures; authors estimated the total cost of self-harm and suicide in New South Wales, Australia as AU$527 million in 2010 and estimated savings due to MATES as AU$3.66 million. The annual budget for MATES was AU$800,000 and therefore the benefit to cost ratio was 6.4:1, i.e. every AU$1 invested produced a return of AU$4.6 [25, 37].

Project expenses in the first year of a peer support programme for clinical and non-clinical staff in one healthcare setting were $12,235 [70]. To set up a wellness programme for medical residents and fellows was estimated to cost $200,000 [71].

Discussion

This scoping review has examined global and cross-industry workplace suicide prevention interventions, describing the mechanisms of interventions by socio-ecological level and synthesising the effectiveness, moderators, implementation barriers and facilitators, and economic costs. Most intervention mechanisms were aimed at a persons' immediate environment and included assessment, treatment, referral and/or crisis support.

Over the past 20 years most studies have been published in the last 10 years. Almost half of the studies in this review were conducted in North America but the remaining studies have global reach, including three LMIC countries. Interventions have been delivered across a variety of sectors, nine in construction and all but one of these related to MATES in Construction. The 12 military studies were across the military (air force, army, special operations, military) whereas 6 of the 11 healthcare studies were aimed at doctors or medical staff. Only three sectors were included in studies that measured effectiveness in terms of rates of suicide: construction, the military and the police.

There have been no randomised controlled trials examining the efficacy of workplace suicide prevention interventions, making it difficult to demonstrate cause and effect. The studies that demonstrated reduced rates of suicide either used before and after study designs or compared rates to other, similar populations. All the studies in this review that did examine rates of suicide found the risk either decreased or rates were lower than comparison populations. This is in line with findings from a review of suicide prevention interventions for emergency and protective services, which found that implementation was associated with suicide rates approximately halving post intervention [12].

This review found that interventions could be successful in changing beliefs and attitudes. There is evidence of the relationship between attitudes towards suicide and suicidal behaviour; having permissive attitudes towards suicide may increase the odds of suicidal ideation, planning and attempts, while viewing suicide as an unjustified behaviour decreases the odds [72]. As demonstrated in this review, attitudes towards suicide can be modified. We did not find any studies that specifically explored the link between modified attitudes and a reduction in suicide behaviours, but evaluations of MATES in Construction have variously reported improvements in attitudes and reduced rates of suicide [e.g. 24, 37]. Further research is needed to examine whether modified attitudes directly lead to reduced suicide behaviours.

Even in industries with rates of suicide higher than the general population, suicide is still rare (ref). This means that to truly assess the impact of organisational suicide intervention programmes, we echo the thoughts of Mishara et al. [66 p.188]:

> Researchers who want to directly assess the impact of programs on deaths by suicide either need to have gigantic budgets to evaluate programs with very large numbers of participants, or they must wait many years, while still offering the program, in order to observe a sufficient number of fatalities to have hopes of obtaining significant findings.

Moreover, we believe that more effort needs to be directed at understanding which elements of such programmes, i.e. mechanisms, provide the greatest impact. Otherwise there is the risk that funds are misdirected away from interventions from the most effective mechanisms towards less effective ones. By combining a realist approach with socio-ecological theory we have attempted to begin to examine this but are hampered by the heterogeneity of the interventions and the studies. For example, the interventions that have demonstrated a reduction in risk or rates of suicide had

little in common in terms of socio-ecological level addressed (ranging from 2 to 5 levels) nor the mechanisms addressing each level. The only common mechanism within the effective interventions was education either aimed at increasing knowledge for individuals or increasing awareness of signs of stress or suicidality in others. However it is unclear what additional mechanisms are required to produce a reduction in risk. For example, the IAM training in the Indian Air Force did not have any mechanisms aimed at the microsystem but did also include peer support and a mentoring programme, which both addressed the mesosystem [73]. In comparison, the US Air Force Suicide Prevention Program did not have any mesosystem level interventions but many aimed at the microsystem: assessment, counselling and crisis support [65]. Both programmes were developed for the air force and both demonstrated positive results. What this review cannot state is which elements are most effective or necessary, nor if there are cultural or procedural factors that potentiate one mechanism over another.

The over-representation of a small number of sectors is unsurprising when considered in the context of population suicide prevalence. Suicide is one of the leading causes of death in men aged 20 to 44 [74]. It is this population that tends to make up the construction workforce as well as military personnel [75, 76] and whilst there are more women registered as doctors or physicians than who work in construction, they are still in the minority [77]. Hence it is not unexpected that there are more workplace suicide prevention initiatives that are designed for industries where rates of suicide amongst workers are likely to be higher. However, while population rates of suicide among women are lower than men [78], rates of suicide amongst women in nursing are higher than female population suicide rates, globally [79-81]. The UK government's suicide prevention strategy highlighted nursing as a high-risk profession for women [82]. Moreover, while the risk of suicide among male medical doctors may be slightly higher than population averages, female doctors' suicide risks are significantly higher [80].

The interventions aimed at healthcare workers in this review had the highest levels of female participants, however none of these studies measured effectiveness in terms of suicide rates and only one identified an improvement in confidence post-intervention [29]. Despite rates of death by suicide being higher among men, women attempt suicide more often; one European study found that two out of three suicide attempt emergency admissions were women [83]. It is important to note that the gender difference may be limited to Western countries; there is little gender difference in rates of suicide in Korea and Japan, for example [84]. The mismatch between rates of suicide attempts and suicide deaths, also known as the 'gender paradox of suicidal behaviour', might be in part because women tend to use less lethal means [85] but other factors, including a higher threshold for help-seeking, may be involved [86]. Increased help-seeking behaviour amongst women appears to be borne out by the findings of this review; the two studies that provided gender data on presentation to services aimed at doctors reported equal numbers of men and women, despite female doctors being in the minority [77]. What is unclear from this review is whether the organisational suicide prevention needs of men and women differ. Suicide risk and protective factors differ by gender, and having an in-depth understanding of the role gender plays in suicidal behaviour is being increasingly recognised as key to improving suicide prevention strategies [87]. Further research is needed to explore organisational suicide prevention activities from a gendered perspective. This may be especially true for high-risk professions such as nursing. Moreover, current research does not factor the impact of intersectionality; in future research it would be beneficial to factor in other characteristics such as ethnicity, sexuality, disability and other protective characteristics in the context of organisational suicide prevention needs.

Conclusions

It is difficult to draw firm conclusions from this review due to the heterogeneity of the studies we identified. It seems likely however, that organisational suicide prevention programmes can have a positive impact on attitudes and beliefs towards suicide as well reducing the risk of suicide. While we cannot, from the evidence, state with any certainty which elements of interventions are likely to be most effective, we can make some informed suggestions. Education, to support individuals to recognise the signs and symptoms of stress, mental ill health and suicidality in both themselves and others, is likely to be an effective starting point for successful interventions. Interventions may need to be tailored to the specific context they are to be implemented in. Nearly all the interventions we identified were developed specifically for the sectors that they were implemented in. This is not to say that there is no possibility of transferring interventions between sectors as the MATES programmes have demonstrated; developed in the construction industry, MATES has been successfully adapted for the energy sector. Similarly, the HEAR programme, developed for medical students, was equally as effective in qualified nurses. Finally, interventions need to be trusted and this may be especially true in sectors where disclosure of health concerns could potentially harm the careers or progression of individuals, such as the military.

Acknowledgements

We appreciate Jordan Omar Blanchard Lafayette's support with title and abstract, and full text screening.

References

1. World Health Organisation. Suicide worldwide in 2019. Global Health Estimates Geneva: World Health Organisation; 2019 [cited 2023 23 January]. Available from: https://www.who.int/publications/i/item/9789240026643.
2. Office for National Statistics. Suicide in England and Wales: 2020 registatrations 2020 [cited 2022 13 July]. Available from: www.ons.gov.uk/peoplepopulationandcommunity/birthsdeathsandmarriages/deaths/bulletins/suicidesintheunitedkingdom/2020registration
3. Solin P, Tamminen N. Scoping review of good practices of suicide prevention in the European region. European Region Journal of Psychological Studies. 2020;2(1):103.
4. van Heeringen K, Mann JJ. The neurobiology of suicide. The Lancet Psychiatry. 2014;1(1):63-72. doi: 10.1016/S2215-0366(14)70220-2.
5. O'Toole M, Mulhall C, Eppich W. Breaking down barriers to help-seeking: preparing first responders' families for psychological first aid. European journal of psychotraumatology. 2022;13(1):2065430-. doi: 10.1080/20008198.2022.2065430. PubMed PMID: 35572389.
6. Health and Safety Executive. Suicide prevention n.d. [cited 2023 10 February]. Available from: https://www.hse.gov.uk/stress/suicide.htm.
7. Causer H, Spiers J, Efstathiou N, Aston S, Chew-Graham CA, Gopfert A, et al. The Impact of Colleague Suicide and the Current State of Postvention Guidance for Affected Co-Workers: A Critical Integrative Review. International journal of environmental research and public health. 2022;19(18):11565. doi: 10.3390/ijerph191811565. PubMed PMID: 36141837.
8. Nigatu YT, Huang J, Rao S, Gillis K, Merali Z, Wang J. Indicated Prevention Interventions in the Workplace for Depressive Symptoms: A Systematic Review and Meta-analysis. American Journal of Preventive Medicine. 2019;56(1):e23-e33. doi: https://doi.org/10.1016/j.amepre.2018.08.027.

9. Joyce S, Modini M, Christensen H, Mykletun A, Bryant R, Mitchell PB, et al. Workplace interventions for common mental disorders: a systematic meta-review. Psychological Medicine. 2016;46(4):683-97. Epub 2015/12/01. doi: 10.1017/S0033291715002408.
10. Orsolini L, Latini R, Pompili M, Serafini G, Volpe U, Vellante F, et al. Understanding the Complex of Suicide in Depression: from Research to Clinics. Psychiatry investigation. 2020;17(3):207-21. Epub 2020/03/23. doi: 10.30773/pi.2019.0171. PubMed PMID: 32209966.
11. Takada M, Shima S. Characteristics and Effects of Suicide Prevention Programs: Comparison between Workplace and Other Settings. Industrial Health. 2010;48(4):416-26. doi: 10.2486/indhealth.MS998.
12. Witt K, Milner A, Allisey A, Davenport L, LaMontagne AD. Effectiveness of suicide prevention programs for emergency and protective services employees: A systematic review and meta-analysis. American Journal of Industrial Medicine. 2017;60(4):394-407. doi: https://doi.org/10.1002/ajim.22676.
13. Peters MDJ, Godfrey C, McInerney P, Munn Z, Tricco AC, Khalil H. Scoping reivews (2020 version). 2020. In: JBI Manual for Evidence Synthesis [Internet]. Available from: https://synthesismanual.jbi.global.
14. Tricco AC, Lillie E, Zarin W, O'Brien KK, Colquhoun H, Levac D, et al. PRISMA extension for scoping reviews (PRISMA-ScR): Checklist and explanation. Annals of Internal Medicine. 2018;169(7):467-73. doi: 10.7326/M18-0850.
15. Wong G, Greenhalgh T, Westhorp G, Buckingham J, Pawson R. RAMESES publication standards: realist syntheses. BMC Medicine. 2013;11(1):21. doi: 10.1186/1741-7015-11-21.
16. Booth A, Briscoe S, Wright JM. The “realist search”: A systematic scoping review of current practice and reporting. Research Synthesis Methods. 2020;11(1):14-35. doi: 10.1002/jrsm.1386.
17. Jagosh J, Pluye P, Wong G, Cargo M, Salsberg J, Bush PL, et al. Critical reflections on realist review: insights from customizing the methodology to the needs of participatory research assessment. Res Synth Methods. 2014;5(2):131-41. Epub 2015/06/09. doi: 10.1002/jrsm.1099. PubMed PMID: 26052652.
18. Johnson SD, Tilley N, Bowers KJ. Introducing EMMIE: an evidence rating scale to encourage mixed-method crime prevention synthesis reviews. Journal of Experimental Criminology. 2015;11(3):459-73. doi: 10.1007/s11292-015-9238-7.
19. Bronfenbrenner U. Developmental Research, Public Policy, and the Ecology of Childhood. Child Development. 1974;45(1):1-5. doi: 10.2307/1127743.
20. Ryan R. Cochrane Consumers and Communication Review Group: Data synthesis and analysis 2013 [cited 2019 20 August]. Available from: http://cccrg.cochrane.org/sites/cccrg.cochrane.org/files/public/uploads/Analysis.pdf.
21. Page MJ, McKenzie JE, Bossuyt PM, Boutron I, Hoffmann TC, Mulrow CD, et al. The PRISMA 2020 statement: an updated guideline for reporting systematic reviews. BMJ. 2021;372:n71. doi: 10.1136/bmj.n71.
22. Nakao M, Nishikitani M, Shima S, Yano E. A 2-year cohort study on the impact of an Employee Assistance Programme (EAP) on depression and suicidal thoughts in male Japanese workers. International Archives of Occupational and Environmental Health. 2007;81(2):151-7. doi: 10.1007/s00420-007-0196-x.
23. King TL, Batterham PJ, Lingard H, Gullestrup J, Lockwood C, Harvey SB, et al. Are Young Men Getting the Message? Age Differences in Suicide Prevention Literacy among Male Construction Workers. International Journal of Environmental Research and Public Health [Internet]. 2019; 16(3).
24. Ross V, Caton N, Gullestrup J, Kõlves K. Understanding the Barriers and Pathways to Male Help-Seeking and Help-Offering: A Mixed Methods Study of the Impact of the Mates in Construction Program. International Journal of Environmental Research and Public Health [Internet]. 2019; 16(16).
25. Doran CM, Ling R, Gullestrup J, Swannell S, Milner A. The Impact of a Suicide Prevention Strategy on Reducing the Economic Cost of Suicide in the New South Wales Construction Industry. Crisis. 2016;37(2):121-9. doi: 10.1027/0227-5910/a000362. PubMed PMID: 26695869.

26. Shelef L, Tatsa-Laur L, Derazne E, Mann JJ, Fruchter E. An effective suicide prevention program in the Israeli Defense Forces: A cohort study. European Psychiatry. 2016;31:37-43. Epub 2020/03/23. doi: 10.1016/j.eurpsy.2015.10.004.
27. Wentworth LM. Suicide prevention and the workplace [PhD]: The University of Iowa; 2016.
28. Davidson JE, Zisook S, Kirby B, DeMichele G, Norcross W. Suicide Prevention: A Healer Education and Referral Program for Nurses. JONA: The Journal of Nursing Administration. 2018;48(2).
29. Willson MN, Robinson JD, McKeirnan KC, Akers JM, Buchman CR. Training Student Pharmacists in Suicide Awareness and Prevention. American Journal of Pharmaceutical Education. 2020;84(8):ajpe847813. doi: 10.5688/ajpe847813.
30. Downs N, Feng W, Kirby B, McGuire T, Moutier C, Norcross W, et al. Listening to Depression and Suicide Risk in Medical Students: the Healer Education Assessment and Referral (HEAR) Program. Academic Psychiatry. 2014;38(5):547-53. doi: 10.1007/s40596-014-0115-x.
31. Garelick AI, Gross SR, Richardson I, von der Tann M, Bland J, Hale R. Which doctors and with what problems contact a specialist service for doctors? A cross sectional investigation. BMC Medicine. 2007;5(1):26. doi: 10.1186/1741-7015-5-26.
32. Rø KEI, Gude T, Aasland OG. Does a self-referral counselling program reach doctors in need of help? A comparison with the general Norwegian doctor workforce. BMC Public Health. 2007;7(1):36. doi: 10.1186/1471-2458-7-36.
33. Warner CH, Appenzeller GN, Parker JR, Warner C, Diebold CJ, Grieger T. Suicide Prevention in a Deployed Military Unit. Psychiatry. 2011;74(2):127-41. doi: 10.1521/psyc.2011.74.2.127.
34. Martin G, Swannell S, Milner A, Gullestrup J. Mates in Construction suicide prevention program: A five year review. Journal of Community Medicine & Health Education. 2016;6:465. doi: 10.4172/2161-0711.1000465.
35. Gullestrup J, Lequertier B, Martin G. MATES in Construction: Impact of a Multimodal, Community-Based Program for Suicide Prevention in the Construction Industry. International Journal of Environmental Research and Public Health [Internet]. 2011; 8(11):[4180-96 pp.].
36. Ross V, Caton N, Gullestrup J, Kõlves K. A Longitudinal Assessment of Two Suicide Prevention Training Programs for the Construction Industry. International Journal of Environmental Research and Public Health [Internet]. 2020; 17(3).
37. Doran CM, Ling R, Gullestrup J, Swannell S, Milner A. The Impact of a Suicide Prevention Strategy on Reducing the Economic Cost of Suicide in the New South Wales Construction Industry. Crisis. 2015;37(2):121-9. doi: 10.1027/0227-5910/a000362.
38. Doran CM, Wittenhagen L, Heffernan E, Meurk C. The MATES Case Management Model: Presenting Problems and Referral Pathways for a Novel Peer-Led Approach to Addressing Suicide in the Construction Industry. International Journal of Environmental Research and Public Health [Internet]. 2021; 18(13).
39. King TL, Gullestrup J, Batterham PJ, Kelly B, Lockwood C, Lingard H, et al. Shifting Beliefs about Suicide: Pre-Post Evaluation of the Effectiveness of a Program for Workers in the Construction Industry. International Journal of Environmental Research and Public Health [Internet]. 2018; 15(10).
40. Ross V, Caton N, Mathieu S, Gullestrup J, Kõlves K. Evaluation of a Suicide Prevention Program for the Energy Sector. International Journal of Environmental Research and Public Health [Internet]. 2020; 17(17).
41. MATES in Construction. The MATES model ND [cited 2022 20 December]. Available from: https://mates.org.au/how-mates-works.
42. Accardi R, Sanchez C, Zisook S, Hoffman LA, Davidson JE. Sustainability and Outcomes of a Suicide Prevention Program for Nurses. Worldviews on Evidence-Based Nursing. 2020;17(1):24-31. doi: https://doi.org/10.1111/wvn.12418.
43. Denzin NK, Lincoln YS, editors. The SAGE handbook of qualitative research. 4th ed. Thousand Oaks, CA: Sage Publications; 2011.

44. Morgan DL. Paradigms lost and pragmatism regained: methodological implications of combining qualitative and quantitative methods. Journal of Mixed Methods Research. 2007;1(1):48-76.
45. Ponterotto JG. Qualitative research in counseling psychology: A primer on research paradigms and philosophy of science. Journal of Counseling Psychology. 2005;52(2):126-36.
46. Lincoln YS, Guba EG. Naturalistic enquiry. Beverly Hills, CA: Sage Publications; 1985.
47. Guba EG. The paradigm dialog. Newbury Park, CA: Sage Publications; 1990.
48. Guba EG, Lincoln YS. Competing paradigms in qualitative research. In: Denzin NK, Lincoln YS, editors. Handbook of qualitative research. 2nd ed. Thousand Oaks, CA: Sage Publications; 1994. p. 105-77.
49. Guba EG, Lincoln YS. Paradigmatic controversies, contradictions, and emerging confluences. In: Denzin NK, Lincoln YS, editors. The Sage handbook of qualitative research. 3rd ed. Thousand Oaks, CA: Sage Publications; 2005. p. 191-215.
50. Lincoln YS, Lynham SA, Guba EG. Paradigmatic controversies, contradictions, and emerging confluences, revisited. In: Denzin NK, Lincoln YS, editors. The SAGE handbook of qualitative research. 4th ed. Thousand Oaks: Sage Publications; 2011. p. 97-128.
51. Howe KR. Against the quantitative-qualitative incompatibility thesis or dogmas die hard. Educational Researcher. 1988;17(8):10-6.
52. Johnson RB, Onwuegbuzie AJ. Mixed methods research: A research paradigm whose time has come. Educational Researcher. 2004;33(7):14-26.
53. Hirsch GA. Nursing: art or science. Canadian Nurse. 1983;79:4-5.
54. Peplau HE. The art and science of nursing: similarities, differences, and relations. Nursing Science Quarterly. 1988;1(1):8-15.
55. Barker P. Psychiatric and mental health nursing: the craft of caring. 2nd ed. London: Hodder Arnold; 2009.
56. Whitley R. Mixed-methods studies. Journal of Mental Health. 2007;16(6):697-701.
57. Kuhn TS. The structure of scientific revolutions. Chicago: University of Chicago Press; 1962.
58. Masterman M. The nature of paradigms. In: Lakatos I, Musgrave A, editors. Criticism and the growth of knowledge. London: Cambridge University Press; 1970.
59. Kuhn TS. The structure of scientific revolutions. 3rd ed. Chicago: University of Chicago Press; 1996.
60. Hoshmand LT. Can lessons of history and logical analysis ensure progress in psychological science? Theory and Psychology. 2003;13(1):39-44.
61. Won GH, Lee JH, Choi TY, Yoon S, Kim SY, Park JH. The effect of a mental health promotion program on Korean firefighters. International Journal of Social Psychiatry. 2020;66(7):675-81. doi: 10.1177/0020764020920918.
62. Kubo H, Urata H, Katsuki R, Hirashima M, Ueno S, Suzuki Y, et al. Development of MHFA-based 2-h educational program for early intervention in depression among office workers: A single-arm pilot trial. PLOS ONE. 2018;13(12):e0208114. doi: 10.1371/journal.pone.0208114.
63. Ramchand R, Saunders J, Osilla KC, Ebener P, Kotzias V, Thornton E, et al. Suicide Prevention in U.S. Law Enforcement Agencies: a National Survey of Current Practices. Journal of Police and Criminal Psychology. 2019;34(1):55-66. doi: 10.1007/s11896-018-9269-x.
64. Finney EJ, Buser SJ, Schwartz J, Archibald L, Swanson R. Suicide prevention in fire service: The Houston Fire Department (HFD) model. Aggression and Violent Behavior. 2015;21:1-4. doi: https://doi.org/10.1016/j.avb.2014.12.012.
65. Knox KL, Pflanz S, Talcott GW, Campise RL, Lavigne JE, Bajorska A, et al. The US Air Force Suicide Prevention Program: Implications for Public Health Policy. American Journal of Public Health. 2010;100(12):2457-63. doi: 10.2105/AJPH.2009.159871.
66. Mishara BL, Fortin L-F. Long-Term Effects of a Comprehensive Police Suicide Prevention Program. Crisis. 2021;43(3):183-9. doi: 10.1027/0227-5910/a000774.

67. Albott CS, Wozniak JR, McGlinch BP, Wall MH, Gold BS, Vinogradov S. Battle Buddies: Rapid Deployment of a Psychological Resilience Intervention for Health Care Workers During the COVID-19 Pandemic. Anesthesia & Analgesia. 2020;131(1).
68. LaCroix JM, Walsh A, Baggett MA, Madison Carter K, the Suicide Care P, Research Initiative T, et al. Three department of defense-funded public health approaches to reduce military suicide. Suicide and Life-Threatening Behavior. 2021;51(2):334-43. doi: https://doi.org/10.1111/sltb.12709.
69. Jimenez C. The Establishment of a Construction Union-Based Member Assistance Program: An Interview With Kyle Zimmer. NEW SOLUTIONS: A Journal of Environmental and Occupational Health Policy. 2021;31(3):350-5. doi: 10.1177/10482911211034173.
70. Adamouski-Marion K. Evaluating the effectiveness of training used for the implementation of a peer support program to support second victims: Saint Francis Medical Center College of Nursuing; 2020.
71. Ey S, Moffit M, Kinzie JM, Brunett PH. Feasibility of a Comprehensive Wellness and Suicide Prevention Program: A Decade of Caring for Physicians in Training and Practice. Journal of Graduate Medical Education. 2016;8(5):747-53. doi: 10.4300/JGME-D-16-00034.1.
72. Kim MJ, Lee H, Shin D, Hong M, Rhee SJ, Park J-I, et al. Effect of Attitude Toward Suicide on Suicidal Behavior: Based on the Korea National Suicide Survey. Psychiatry Investig. 2022;19(6):427-34. doi: 10.30773/pi.2021.0361.
73. Joseph C, Roopa CG, Kumar U, Bhatti RS, Panhasarathy R, Chandramohan V, et al. Prevention of suicide: The IAM awarence training programme. Indian Journal of Aerospace Medicine. 2004;48(2):8-16.
74. Centers for Disease Control and Prevention. Leading Causes of Death – Males – All races and origins – United States, 2018 2018 [cited 2023 2 January]. Available from: https://www.cdc.gov/minorityhealth/lcod/men/2018/all-races-origins/index.htm.
75. House of Commons Defence Committee. Protecting those who protect us: Women in the Armed Forces from Recruitment to Civilian Life 2021 [cited 2023 2 January]. Available from: https://committees.parliament.uk/publications/6959/documents/72771/default/.
76. Munn M. Building the future: Women in construction London: The Smith Institute; 2014 [cited 2023 2 January]. Available from: https://women-into-construction.org/wp-content/uploads/2019/08/building-the-future-women-in-construction.pdf.
77. Begeny CT, Grossman RC, Ryan MK. Overestimating women's representation in medicine: a survey of medical professionals' estimates and their(un)willingness to support gender equality initiatives. BMJ Open. 2022;12(3):e054769. doi: 10.1136/bmjopen-2021-054769.
78. Baker C. Suicide statistics London: House of Commons Library; 2022 [cited 2023 2 January]. Available from: https://researchbriefings.files.parliament.uk/documents/CBP-7749/CBP-7749.pdf.
79. The National Confidential Inquiry into Suicide and Safety in Mental Health (NCISH). Suicide by female nurses: A brief report. Manchester: University of Manchester, 2020.
80. Case R, Alabakis J, Bowles K-A, Smith K. Suicide prevention in high-risk occupations: an Evidence Check rapid review brokered by the Sax Institute New South Wales: Sax Institute; 2020 [cited 2023 2 January]. Available from: https://www.saxinstitute.org.au/wp-content/uploads/20.10_Evidence-Check_Suicide-prevention-in-high-risk-occupations.pdf.
81. Peterson C, Sussell A, Li J, Schumacher PK, Yeoman K, Stone DM. Suicide rates by industry and occupation - National Violence Death Reporting System, 32 States. MMWR Morb Mortal Wkly Rep. 2016;69:57-62. doi: http://dx.doi.org/10.15585/mmwr.mm6903a1.
82. HM Government. Preventing suicide in England: Fourth progress report of the cross-government outcomes strategy to save lives. London: 2019.
83. Lopez-Castroman J, Perez-Rodriguez MdlM, Jaussent I, Alegria AA, Artes-Rodriguez A, Freed P, et al. Distinguishing the relevant features of frequent suicide attempters. Journal of Psychiatric Research. 2011;45(5):619-25. doi: https://doi.org/10.1016/j.jpsychires.2010.09.017.

84. Hee Ahn M, Park S, Ha K, Choi SH, Hong JP. Gender ratio comparisons of the suicide rates and methods in Korea, Japan, Australia, and the United States. Journal of Affective Disorders. 2012;142(1):161-5. doi: https://doi.org/10.1016/j.jad.2012.05.008.
85. Hegerl U. Prevention of suicidal behavior. Dialogues in Clinical Neuroscience. 2016;18(2):183-90. doi: 10.31887/DCNS.2016.18.2/uhegerl.
86. Cibis A, Mergl R, Bramesfeld A, Althaus D, Niklewski G, Schmidtke A, et al. Preference of lethal methods is not the only cause for higher suicide rates in males. Journal of Affective Disorders. 2012;136(1):9-16. doi: https://doi.org/10.1016/j.jad.2011.08.032.
87. Samaritans. Research briefing: Gender and suicide 2021 [cited 2023 6 January]. Available from: https://media.samaritans.org/documents/ResearchBriefingGenderSuicide_2021_v7.pdf.

Figure
Click here to access/download;Figure;Organisational suicide prevention scoping review_Figure 1.docx

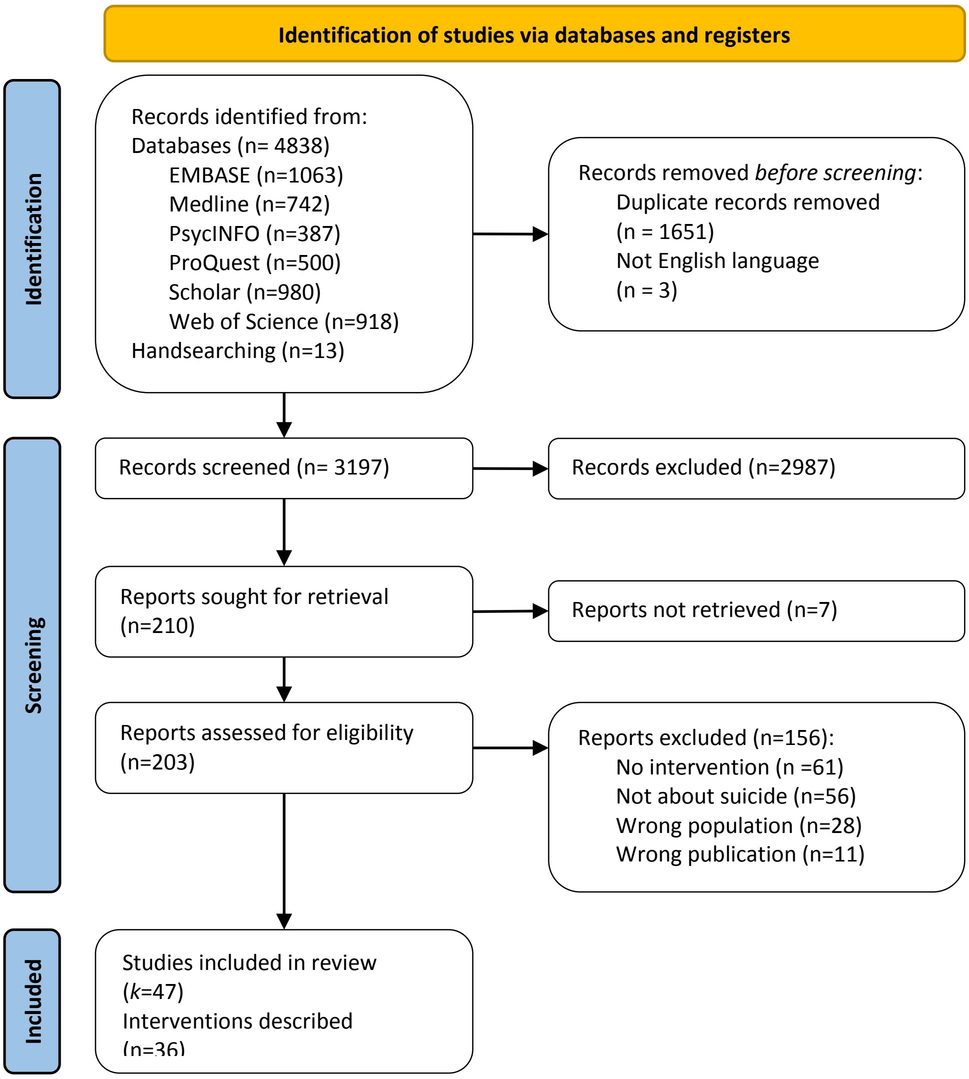

Fig 1. PRISMA flow diagram [21]

Supplementary tables

Click here to access/download
Supporting Information
Organisational suicide prevention_Supplementary.docx

Click here to access/download
Supporting Information
Organisational suicide prevention_PRISMA-ScR checklist.docx
